# Demystifying the manipulation of host immunity, metabolism, and extraintestinal tumors by the gut microbiome

**DOI:** 10.1038/s41392-019-0074-5

**Published:** 2019-10-12

**Authors:** Ziying Zhang, Haosheng Tang, Peng Chen, Hui Xie, Yongguang Tao

**Affiliations:** 1 Key Laboratory of Carcinogenesis and Cancer Invasion, Ministry of Education, Department of Pathology, Xiangya Hospital, Central South University, 410078 Hunan, China; 2 NHC Key Laboratory of Carcinogenesis (Central South University), Cancer Research Institute and School of Basic Medicine, Central South University, 410078 Changsha, Hunan China; 3 Hunan Key Laboratory of Tumor Models and Individualized Medicine, Department of Thoracic Surgery, Second Xiangya Hospital, Central South University, 410011 Changsha, China; 4 Department of Oncology, Third Xiangya Hospital, Central South University, 410013 Changsha, China; 5 Department of Urology, Xiangya Hospital, Central South University, 410008 Changsha, China; 6 Department of Thoracic and Cardiovascular Surgery, Second Xiangya Hospital of Central South University, 410011 Changsha, China

**Keywords:** Cancer, Cancer therapy, Cancer metabolism

## Abstract

The trillions of microorganisms in the gut microbiome have attracted much attention recently owing to their sophisticated and widespread impacts on numerous aspects of host pathophysiology. Remarkable progress in large-scale sequencing and mass spectrometry has increased our understanding of the influence of the microbiome and/or its metabolites on the onset and progression of extraintestinal cancers and the efficacy of cancer immunotherapy. Given the plasticity in microbial composition and function, microbial-based therapeutic interventions, including dietary modulation, prebiotics, and probiotics, as well as fecal microbial transplantation, potentially permit the development of novel strategies for cancer therapy to improve clinical outcomes. Herein, we summarize the latest evidence on the involvement of the gut microbiome in host immunity and metabolism, the effects of the microbiome on extraintestinal cancers and the immune response, and strategies to modulate the gut microbiome, and we discuss ongoing studies and future areas of research that deserve focused research efforts.

## Introduction

The gut microbiome (and its collective genomes, namely, the microbiome) is composed of trillions of bacteria, archaea, viruses, fungi, and other microeukaryotic colonizers.^1^ It is estimated that 3 × 10^13^ bacteria reside in the human gut, which is close to the number of cells in the human body.^2^ Four primary microbial phyla, including *Firmicutes*, *Bacteroides*, *Proteobacteria*, and *Actinobacteria*, comprise 98% of the gut microbiome in healthy adults, of which *Firmicutes* (60–80%) and *Bacteroides* (15–25%) are the dominant bacterial species. The diversity and density of microbial species increases longitudinally from the stomach to the colon, where the microbiome community (over 10^13^ microbial cells) is the most abundant and metabolically exuberant.^3^ Shockingly, the human microbiome contains over 3 million genes,^4^ a staggering number, especially when one considers that there are only 20,000–25,000 genes in the human genome.^5^ Approximately 60–80% of the gut microbiome cannot be cultivated under laboratory conditions; thus, much of the genome sequences of these species remain unknown. One of the culture-independent approaches is the reestablishment of metagenome-assembled genomes from human gut microbiomes, which has identified ~2500 previously unknown species and increased the diversity of the known bacterial repertoire to more than 4500 species.^6^ Another study used a similar research method to identify nearly 2000 uncultured candidate bacterial species, substantially increasing the bacterial phylogenetic diversity.^7^ Additionally, over 7000 microbial genomic structural variants (SVs) have been identified thus far in the human gut microbiome, and they have shown an association with disease risk factors. For example, a variant region in *Anaerostipes hadrus* encodes the biosynthesis of butyrate to decrease the risk of metabolic disease in the host, potentially explaining the difference in body weight between individuals carrying such microbial SVs and those who do not.^8^

The dynamic functional network composed of the gut microbial ecosystem, systemic metabolism, and immune system is of extraordinary significance to realize and maintain host health and homeostasis. The gastrointestinal tract confers a natural anaerobic environment conducive to colonization.^9^ Reciprocally, the gut microbiome exerts important effects on host physiology, including controlling post-translational modifications of the host proteome,^10^ stimulating immune system development and homeostasis,^11,12^ maintaining intestinal barrier integrity,^13^ reaping inaccessible nutrients from the diet,^14^ synthesizing certain essential vitamins and neurotransmitters,^15^ modulating neurobehavioral properties,^16,17^ endocrine functions^18^ and bone density,^19^ and even participating in drug biotransformation.^20,21^

Multiple factors can lead to a loss of beneficial microbes and a reduction in microbial diversity, ultimately triggering gut dysbiosis (microbial imbalance or maladaptation). A wide range of studies have revealed the potential role of gut dysbiosis in many human diseases. It can mediate intestinal metabolic functions, mucosal inflammation, and immunity through local effects and has profound effects on gastrointestinal disorders, including inflammatory bowel disease (IBD)^22^ and colorectal carcinoma.^23^ It can also impact extraintestinal organs in distant parts of the body through diversiform and distinct mechanisms, including the translocation of the gut microbiome or/and their structure and components, the circulation of microbial-derived metabolites or endocrine molecules, the migration of immune cells and factors, and the modulation of gut–brain axis signaling through the vagal nerve, leading to neuropsychiatric diseases (depression, autism),^16,24^ autoimmune diseases (autoimmune diabetes, systemic lupus erythematosus, and allergies),^25–27^ metabolic diseases (obesity, type 2 diabetes, nonalcoholic fatty liver),^28–31^ and even extraintestinal tumors (hepatocellular carcinoma, breast cancer, pancreatic cancer, and melanoma).^32–35^ Notably, there is a wide array of evidence that microbial metabolites derived from ingested nutrients (such as short-chain fatty acids (SCFAs), microbial tryptophan (TRP) catabolites, and succinate) are pivotal inducers of such effects.

The mammalian intestine serves as a fertile ground where host–microbiota interactions occur. The gut commensals that establish harmonious relationships with the host are essential for the development and appropriate function of the immune system via metabolite-independent mechanisms. The gut microbiome is an effective stimulator of the immune response in the gut.^36,37^ However, environmental exposure and genetic deficits in combination with gut dysbiosis potentially contribute to the manifestation of host immunity disorders and various inflammatory diseases.^38–40^ Correspondingly, immune signals induced by the gut microbiome in turn function as a powerful weapon to modulate gut commensals^41,42^ and to protect against pathogen invasion.^43^ It is essential to understand the perplexing and reciprocal interaction between the gut microbiome and host immune system, especially effects on the differentiation of regulatory T cells (Treg cells), T helper 17 (Th17) cells, and T helper 1 (Th1) cells that account for the majority of effector T (Teff) cells in the gut and immunoglobulin A (IgA)-producing B cells, as well as group 3 innate lymphoid cells (ILC3s).

## Microbial metabolite-mediated modulation of host immunity and metabolism

### Gut microbial SCFAs

Certain intestinal anaerobic bacteria, specifically the members of the *Clostridium* genus, such as cluster IV (*Faecalibacterium prausnitzii*^44^) and cluster XIVa (*Anaerostipes butyraticus*^45^ and *Roseburia intestinalis*,^46^) harbor the capability to convert indigestible carbohydrates into fermentation products, including SCFAs (particularly acetate, propionate, and butyrate).^47^ The concentration of SCFAs varies longitudinally in the intestine, with a peak level in the cecum and proximal colon.^48^

SCFAs (especially butyrate) can be absorbed into colonocytes via passive transport, SLC5A8-dependent transit, or the recognition of G protein-coupled receptors (GPCRs or GPRs) to function as energy sources.^48^ They are also transferred through the portal vein to the liver, and a residual amount that is unextracted and unmetabolized by the liver reaches the systemic circulation to regulate peripheral organs.^48^ Below, we unveil the intricate and dynamic interaction among SCFAs, the host immune system, and metabolism, which is instrumental in ameliorating the corresponding deficits and contributing to host homeostasis (Supplementary Table 1).

### Microbially derived SCFAs mitigate gut inflammation

**Fig. 1:**
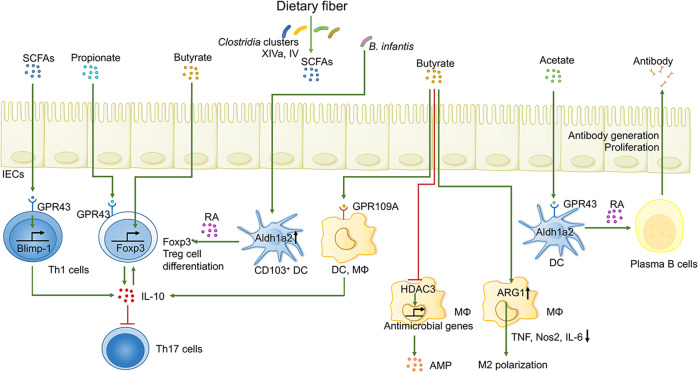
Mechanisms of signaling from microbial-derived SCFAs to multiple immune cells in the gut. SCFAs participate in a sophisticated and dynamic host–microbiome network to orchestrate intestinal immune responses (such as Treg development, macrophage and DC activity, and the release of anti-inflammatory cytokines or AMP, plasma B cell proliferation, and antibody production) by suppressing HDAC or by stimulating GPRs (such as GPR109A and GPR43), ultimately exerting anti-inflammatory effects and conferring resistance against pathogens

SCFAs can act on various immune cells in the gut to inhibit inflammation through multiple mechanisms (Fig. 1). The differentiation of anti-inflammatory forkhead box protein P3 (Foxp3)^+^ Treg cells can be modulated by SCFAs.^49^ Initially, by acting through GPR43 (also known as free fatty acid receptor 2, FFAR2), propionate stimulates interleukin-10 (IL-10)-producing Foxp3^+^ Treg cell differentiation and thus protects against experimental colitis.^50,51^ SCFA-mediated GPR43 signaling also elicits NLRP3 inflammasome activation and the resulting IL-18 secretion to control barrier integrity^52,53^ and was recently revealed to protect against gastrointestinal graft-versus-host disease (GvHD).^54^ Similarly, butyrate binds GPR109A on intestinal dendritic cells (DCs) and macrophages, fostering an IL-10-rich and class 1A aldehyde dehydrogenase (Aldh1a)-rich environment, which boosts Treg cell development while inhibiting proinflammatory Th17 cell expansion.^55^ Second, butyrate is well recognized as a histone deacetylase (HDAC) inhibitor, and the suppressive effect of butyrate on HDAC occurs in part by tightly binding to Zn^2+^ in the active site of HDAC.^56^ Butyrate increases the acetylation of histone H3 at the Foxp3 promoter and at the enhancer conserved noncoding sequence 1 (CNS1), ultimately eliciting robust gene expression and functional maturation.^57,58^ Butyrate derived from commensal bacteria *Clostridium* exerts epigenetic control over transforming growth factor β (TGF-β) in intestinal epithelial cells (IECs), a process mediated by its HDAC-inhibitory activity and through transcription factor specific protein binding on the core promoter, which drives TGF-β1 expression in IECs and the subsequent convergence of Treg cells in the intestine.^59^ Moreover, TGF-β in conjunction with retinoic acid (RA) generated from Aldh1a2-expressing DCs facilitates the development of Foxp3^+^ Treg cells.^60^ Through this process, the Foxp3 gene intronic enhancer CNS1 is endowed with a combined location for the RA receptor, supporting RA-mediated Foxp3^+^ pTreg cell development.^61^ Furthermore, symbiont *Bifidobacterium infantis* (*B. infantis*) is sufficient to enhance the number of CD103^+^ DCs and potentiate their capability to generate RA in the gut.^62^ Further studies are required to address what additional intestinal cell types or transcription factors respond to SCFA-mediated HDAC-inhibitory activity to orchestrate intestinal immunity. Collectively, these results demonstrate the profound function of SCFAs in the development of Treg cells.

Accumulating evidence has provided novel insights into the underlying mechanisms by which host–SCFA crosstalk exerts immunomodulation to Teff cells. If the host is in the context of combating pathogens, SCFAs will stimulate the differentiation of Th1 cells and protective Th17 cells to enhance immunity. For example, during *Citrobacter rodentium* (*C. rodentium*) infection, the administration of acetate induces the differentiation of Th1 and Th17 cells directly through its HDAC inhibitor activity rather than through GPR43 or GPR41, leading to increased acetylation of p70S6 kinase and phosphorylation of rS6, and thus modulation of the mammalian target of rapamycin (mTOR) pathway, which is a prerequisite for Th17 and Th1 cell development.^63^ SCFAs might facilitate IL-10 generation by microbiome antigen-specific Th1 cells through the GPR43 signaling pathway. Mechanistically, SCFAs favor the expression of B lymphocyte-induced maturation protein 1 (Blimp-1) in Th1 cells by activating STAT3 and mTOR pathways, thereby accelerating IL-10 generation by Th1 cells and alleviating colitis in mice.^64^ Moreover, butyrate-induced IL-10 production is also modulated by Blimp-1 during Th1 cell differentiation.^65^ Butyrate signals through GPR41 and GPR43 to accelerate the metabolism of antigen-activated CD8^+^ T cells, thereby enhancing their memory potential.^66^ These findings highlight that SCFAs can induce T cell development into both Teff cells and Treg cells to drive either anti-pathogen immunity or immune tolerance on the basis of the immunological milieu.

SCFAs are also immunopotentiators to enhance antibody production in the gut lumen, benefiting the host. SCFAs initiate metabolic processes in B cells to support antibody production, including facilitating the synthesis of acetyl-CoA, adenosine 5′-triphosphate (ATP), and fatty acids, boosting energy, and increasing the number of building blocks.^67^ Additionally, SCFAs modulate gene expression through their HDAC inhibitor activity to enhance the expression of key genes (such as *Xbp1*, *Irf4*, and *Aicda*) for both local and systemic plasma cell (PC) differentiation.^67,68^ SCFAs also stimulate the production of BAFF and Aldh1a2 by DCs to upregulate plasma B cell differentiation-related genes.^69^ These antibodies accelerate pathogen elimination while facilitating the colonization of certain gut-resident commensals.^70,71^ DCs can act as pivotal intermediaries for SCFA-mediated IgA production in B cells. Acetate-mediated GPR43 signaling on intestinal DCs indirectly potentiates IgA generation by B cells, a manipulation dependent on diversified pathways, including the generation of DC-derived RA and activation of the mTOR pathway in DCs.^72^ SCFAs have also been shown to signal through GPR43 to promote intestinal antibody responses elicited by cholera toxin (CT), highlighting the critical role of SCFAs in promoting mucosal adjuvant activity of CT.^69^ Therefore, the generation and release of antibodies into the intestinal tract partly depend on the perception and recognition of SCFAs.

SCFAs act through multiple distinct mechanisms to modulate the activities of intestinal macrophages. For example, the suppressive effect of butyrate on HDAC3 drives anti-microbial gene expression, further boosting anti-microbial peptide (AMP) production, such as S100A8/A9/A12 and lysozyme, consequently bolstering enteropathogen clearance.^73^ Similarly, the exposure of bone marrow (BM)-derived macrophages to *n*-butyrate abrogates the release of lipopolysaccharide (LPS)-induced proinflammatory cytokines, including nitric oxide (NO), IL-6, and IL-12, by enhancing acetylation of the promoter regions of these corresponding genes and reducing subsequent gene transcription.^74^ Additionally, treating colonic macrophages with butyrate decreases the production of tumor necrosis factor (TNF) protein.^75^ Butyrate triggers a metabolic shift in macrophages from glycolysis to oxidative phosphorylation and lipid metabolism, which is dependent on the upregulation of Arg1 expression^75,76^ and the inhibition of HDAC3 activity,^73^ thereby favoring macrophage polarization towards an anti-inflammatory M2 phenotype.^73,76^ Conversely, antibiotic-mediated gut dysbiosis and SCFA depletion may facilitate the expansion of proinflammatory Th1 cells through the activation of proinflammatory macrophages, contributing to susceptibility to infection.^75^ These findings highlight that the SCFA-mediated anti-inflammatory function is partially dependent on M2 macrophages. However, whether these SCFA-mediated functional alterations in intestinal macrophages are GPR-dependent remain unclear.

### SCFAs confer colonization resistance against intestinal pathogens

**Fig. 2:**
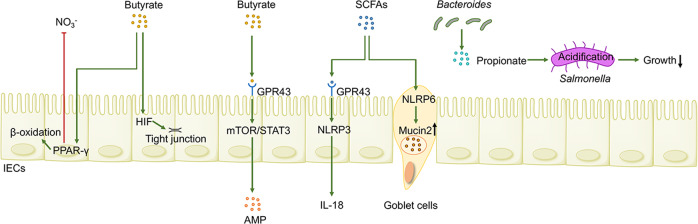
Gut microbiome-associated SCFAs shape the homeostatic host–microbiome interface. SCFAs foster a hypoxic microenvironment by activating PPAR-γ and undermining the pH homeostasis of pathogens to inhibit pathogen growth. SCFAs also signal through GPRs (such as GPR43) to secrete IL-18 and AMP, contributing to enhanced intestinal barrier function

IECs function as gatekeepers of the innate immune system and affect the intestinal microenvironment following the identification of and response to microbial-derived SCFA irritation (Fig. 2).^77^ SCFAs participate in regulating the colonic metabolic state to foster an intestinal environment conducive to commensals. Under gut homeostatic conditions, the butyrate-mediated activation of peroxisome proliferator-activated receptor gamma (PPAR-γ, nuclear receptor primarily synthesized in IECs) promotes the mitochondrial β-oxidation of SCFAs as well as oxidative phosphorylation in colonocytes, thereby maintaining a local hypoxic microenvironment. The obligate anaerobic SCFA-producing bacteria thrive while the overgrowth of facultative anaerobic enteric pathogens such as *Escherichia coli* (*E. coli*, a surrogate marker for dysbiosis) and *Salmonella* is suppressed in such conditions.^78,79^ Simultaneously, PPAR-γ activation suppresses Nos2 expression in IECs as well as the production of inducible NO synthase (an enzyme that produces NO) and nitrate (a crucial energy source for facultative anaerobic pathogens).^80^ Additionally, *Bacteroides*-derived propionate has been shown to confer colonization resistance to pathogens in a PPAR-γ-independent manner, suggesting the functional redundancy present in SCFAs. Indeed, propionate facilitates the cytoplasmic acidification of *Salmonella* and disrupts the intracellular pH homeostasis of pathogens, thereby limiting pathogen expansion.^81^ Indeed, the functional metabolic capabilities of certain commensals confer protection against pathogen infection, which is attributable to the intracellular acidification of pathogens mediated by SCFAs.^82^ High concentrations of SCFAs and the acidic environment reverse or counteract the competitive advantage that O_2_ and NO_3_ respiration provide to facultative anaerobes such as *Enterobacteriaceae*.^82^ Conversely, antibiotic treatment elicits gut dysbiosis and SCFA exhaustion, which further inhibits the PPAR-γ signaling pathway and induces metabolic reprogramming. This reprogramming shifts colonocytes towards anaerobic glycolysis and away from oxidative metabolism, which markedly elevates the levels of oxygen and nitrate as well as lactate in the gut lumen, thus driving *Enterobacteriaceae* expansion.^78,83^ Moreover, elevated levels of *Salmonella* (family *Enterobacteriaceae*) utilize virulence factors to induce neutrophil transepithelial migration, contributing to the depletion of *Clostridia* and diminished concentrations of SCFAs. This negative feedback loop creates an environment that is more conducive to pathogen colonization.^79^ These findings have unveiled a causal interplay between microbiota-derived SCFAs and metabolism in the gut epithelium, setting the stage for the development of microbial and metabolite-based drugs for clinical translation and, potentially, therapies targeting PPAR-γ.^61^

### Microbially derived SCFAs enhance gut barrier integrity

SCFAs have also emerged as an important regulator of a physio-chemical barrier to support the integrity of the gut mucosal barrier by stimulating AMPs and mucus generation by Paneth cells and goblet cells, respectively (Fig. 2).^84^ By acting through specific GPCRs, SCFAs potentially activate NLRP6 to facilitate intestinal goblet cells to secrete Mucin2.^85–87^ *Clostridia*-derived butyrate alleviates GvHD by potentiating IEC proliferation and apical junctional protein expression through HDAC inhibition.^88^ Butyrate elicits anti-inflammatory IL-10 receptor-α subunit by activating STAT3 and inhibiting HDAC, which increases the production of colonic Mucin2 and tight junction proteins and consequently protects against LPS leakage and inflammation.^89,90^ Butyrate also binds to GPR43 on IECs to generate AMPs such as RegIIIγ and β-defensin by activating mTOR and STAT3.^91^ The synergistic effect between butyrate-induced AMP cathelicidin and mucus formation confers to the host an optimal innate response against amebic colitis.^92^ Butyrate activates the transcription factor hypoxia-inducible factor-1 (HIF-1) in IECs, thereby protecting the intestinal barrier from damage caused by *Clostridium difficile* (*C. difficile*) toxins.^93^ In the setting of dietary fiber deficiency, the gut microbiome preferentially utilizes mucins or polysaccharides as energy sources, which undermines the permeability of the internal mucus layer and compromises the spatial separation between gut commensals and the intestinal lamina propria, thus predisposing the gut to the invasion of pathogen *C. rodentium*, indirectly proving the importance of SCFAs in the intestinal chemical barrier.^94–96^ Total SCFA levels were found to be dramatically diminished in a high-fat diet (HFD) group (fat 40% energy) than in low-fat or moderate-fat diet groups, supporting the significance of a high-fiber diet and low-fat diet in keeping adequate SCFA levels and long-term fitness.^97^ Notably, the exposure of stem cells to a high concentration of butyrate by mucosal injury results in butyrate-mediated HDAC inhibition and impaired stem cell function, which potentially exerts detrimental impacts on intestinal regeneration and wound repair in a colitis model.^98^ However, stem cell proliferation is also impeded when in contact with potentially pernicious components in the lumen because of this appropriately inhibitory action of butyrate.^98^ Moreover, oral administration of inulin exacerbates αIL10R-induced colitis, which is largely attributable to the enrichment of butyrate-producing bacteria and elevated levels of cecal butyrate, indicating that the overproduction of SCFAs may exert detrimental effects on the host.^99^

Collectively, SCFAs are considered the most abundant microbiome-derived metabolites in the gut lumen and are endowed with the robust capacity to dampen intestinal inflammation, protect against pathogen invasion and maintain barrier integrity largely by activating GPCRs or inducing their suppressive effects on HDACs to further influence gene expression.

### SCFA-mediated modulation of host metabolism

Compelling and accumulating evidence has addressed both the association and the causality between microbiota-derived SCFAs and metabolic disorders in animal models of obesity or metabolic diseases.^100,101^ Additionally, fecal SCFA levels are significantly decreased in healthy young adults following long-term (6 months) HFD.^97^ Dietary fiber facilitates significant enrichment of a select group of SCFA-producing strains in patients with type 2 diabetes. Moreover, the higher the abundance and diversity of SCFA-producing bacteria are, the more improvement observed in the hemoglobin A1c levels of the subjects, which can be partly attributed to the SCFA-mediated increase in glucagon-like peptide-1 (GLP-1) production.^47^ However, this study is not sufficient to highlight the causal metabolic links between certain SCFAs and type 2 diabetes. Some methodologies, such as genome-wide genotyping, gut metagenomic sequencing, and fecal SCFA level analysis, are powerful when utilized in conjunction with one another and demonstrated that butyrate is capable of improving insulin response, while deficiencies in the generation or utilization of propionate enhance the risk of type 2 diabetes.^102^

Mechanically, SCFAs prevent obesity by modulating appetite and energy intake. First, SCFAs stimulate the generation of anorectic hormones. Both animal and human studies have revealed that enhanced levels of acetate and butyrate in the intestinal lumen stimulate enteroendocrine L cells to produce GLP-1 and fasting peptide YY (PYY), leading to a significant reduction in food intake.^47,103–105^ In addition, the inhibition of energy intake by SCFAs is partly dependent on central nervous system (CNS)-related mechanisms and the gut–brain axis. Indeed, vagal afferent chemoreceptors potentially sense SCFAs or gut hormones such as GLP-1 to presumably dominate the acervulus cerebri and eventually decrease appetite.^106^ Acute oral administration of butyrate restricts the activity of neuropeptide Y-expressing orexigenic neurons and eventually drives satiety and diminished appetite.^107^ Elevated levels of acetate elicit anorectic effects in the hypothalamus, possibly via inhibiting 5-AMP-activated kinase.^108,109^ However, another group has drawn the opposite conclusion that the increased levels of acetate in rodents on a HFD activate the parasympathetic nervous system and thus upregulate ghrelin and glucose-stimulated insulin secretion to trigger hunger, insulin resistance, and hypertriglyceridemia.^110^ Alternatively, microbiome-derived acetate seems to potentiate liver glucogenesis, ultimately supporting the development of metabolic syndrome in toll-like receptor 5 (TLR5)-deficient mice.^111^ Thus, the effects of acetate on host metabolism require further investigation.

Furthermore, butyrate and propionate stimulate intestinal gluconeogenesis (IGN) to maintain glucose and energy homeostasis. More precisely, butyrate upregulates IGN gene expression, which is dependent on a cAMP-mediated mechanism. However, propionate, itself a substrate of IGN, signals through GPR41 in the periportal afferent neural system to stimulate IGN.^112^

SCFAs also exert beneficial effects on host metabolism and weight control by increasing energy expenditure and lipid oxidation. In mice with HFD-induced obesity, the administration of butyrate leads to remarkable decreases in body weight, mainly driven by higher energy expenditure and increased catabolism. Such an effect is associated with the increased expression of genes that regulate metabolism (such as Pck1) and the significantly decreased activity of HDAC3, which is involved in promoting obesity.^113^ Butyrate administration shifts enterocyte metabolism from glycolysis towards fatty acid utilization, thereby mitigating the development of endotoxemia and atherosclerosis in mice.^114^ Acetate also serves as a microbial metabolic signal to activate the immune deficiency innate immune pathway in enteroendocrine cells in the *Drosophila* model, thereby facilitating the production of the endocrine peptide Tachykinin, which is imperative for optimal lipid metabolism.^115^ Interestingly, the thermogenic capacity of brown adipose tissue is drastically impaired in mice treated with antibiotics, a phenotype that is counteracted by gavage of butyrate, providing a novel avenue through which to demonstrate the correlation between the gut microbiome and its metabolites with thermoregulation.^116^ It remains to be investigated whether these benefits from SCFA oxidative metabolism will be reflected in humans. Intriguingly, the lactate produced by exercise enters the gut through the circulatory system and specifically enhances the growth of *Veillonella*, which can catabolize lactate into propionate, thereby enhancing the host’s athletic performance.^117^ Therefore, SCFAs exert beneficial effects on host metabolism and ameliorate metabolic disorders, largely by modulating dietary behavior and energy expenditure in the host, indicating the significance of SCFAs in the gut–brain axis.

### SCFA-mediated immunoregulation in extraintestinal diseases

SCFAs can enter the systemic circulation to facilitate additional crosstalk between the extraintestinal tissues and the gut. Specific examples include the following.

First, SCFAs modulate the gut–brain axis. SCFA administration is sufficient to abolish the microglial maturation defects observed in germ-free (GF) mice.^118^ Similarly, butyrate suppresses cuprizone-induced demyelination in oligodendrocyte precursor cells and accelerates oligodendrocyte differentiation,^119^ unveiling the effect of SCFAs on CNS immune cell homeostasis. Additionally, treatment with butyrate in ischemic stroke models effectively enriches the levels of *Lactobacillus* and restores the leaky gut.^120^ SCFAs are known to maintain blood–brain barrier (BBB) integrity by upregulating the tight junction protein occludin.^121^ Bacteria-derived propionate functions as a ligand for the brain-endothelium-expressed FFAR3 to suppress TLR pathways related to nonspecific microbial infections through a CD14-mediated manner,^122^ highlighting that the effects of SCFAs on barrier integrity are not confined to only the gut mucosal barrier.^122^ Supplementation with SCFAs activates NLRP6 and further restores the impaired gut mucosal barrier, thereby suppressing high-fructose diet-triggered hippocampal neuroinflammation and neuronal deficiency.^123^ These findings demonstrate the remarkable role of SCFAs in ameliorating CNS inflammation.

Additionally, impaired insulin production and aberrant insulin distribution in pancreatic β-cells are observed in GF mice.^124^ Impaired gut barrier integrity contributes to the activation of islet-specific T cells in the intestinal mucosa and in autoimmune diabetes.^125^ These findings emphasize the association between the gut microbiome and autoimmune diabetes. Healthy infants harbor much higher levels of genes associated with bacterial fermentation and SCFA biosynthesis in the gut than do participants with type 1 diabetes, and SCFAs potentially play a protective role in early-onset type 1 diabetes.^126^ SCFAs have been shown to mitigate insulitis and type 1 diabetes in nonobese diabetes (NOD) mice through multiple distinct mechanisms.^127,128^ Acetate can inhibit autoreactive T cells by diminishing the levels of marginal zone B cells, whereas butyrate can enhance the number and function of Foxp3^+^ Treg cells by increasing Foxp3 locus acetylation to maintain self-tolerance.^127^ Oral supplementation with butyrate in NOD mice confers protection against autoimmune diabetes by promoting the proliferation of pancreatic immunosuppressive macrophages and Treg cells.^128^ The ability of SCFAs derived from a special starch diet to restrain the abundance and translocation of *Lactobacillus* *reuteri* (*L. reuteri*), a bacterium that potentiates plasmacytoid DCs and type I interferon (IFN) pathways to enable the development of autoimmune manifestations, is sufficient to ameliorate systemic lupus erythematosus.^129^

Furthermore, a higher abundance of butyrate-producing microbiota in fecal bacteria correlates with enhanced protection against respiratory viral infection with lower respiratory tract infections in allogeneic hematopoietic stem cell transplantation patients.^130^ Acetate also signals through GPR43 and IFN-1 receptor (IFNAR) on pulmonary epithelial cells and further stimulates IFN-β response, thus conferring protection against respiratory syncytial virus (RSV) infection.^131^ SCFAs also protect against influenza infection and dampen deleterious tissue immunopathology through two complementary mechanisms. SCFAs activate macrophages with the capacity to alleviate neutrophil-mediated tissue damage. Simultaneously, SCFAs boost anti-viral CD8^+^ T cell function by augmenting their cellular metabolism.^132^

SCFAs are also potent regulators of osteoclast metabolism and bone homeostasis via the gut–bone axis. For example, supplementation with *Lactobacillus rhamnosus GG* (*LGG*) in mice enhances both local and systemic butyrate that supports the expansion of Treg cells in the gut and BM. Treg-derived TGF-β in the BM facilitates the release of Wnt10b by BM-resident CD8^+^ T cells and thereby stimulates bone anabolism and bone homeostasis.^19^ Propionate and butyrate trigger metabolic reprogramming that shifts osteoclasts towards enhanced glycolysis at the expense of oxidative phosphorylation, thereby preventing pathological bone loss.^133^

Hence, the benefits of SCFAs on the host are not limited in the gut, as they can disseminate into the bloodstream and thus communicate with multiple cells in target tissues in a GPCR-dependent manner or by suppressing HDAC activity. Notably, the effects of microbially derived SCFAs are potentially context-dependent. SCFAs are generally beneficial during homeostasis while exerting deleterious effects in the context of inflammation. Additionally, the dose, duration, and host genetics are the determinants of whether intestinal SCFAs trigger physiological or pathological effects.^32,99,111^

### Microbially derived TRP metabolites

Certain aspects of the gut microbiome can convert food components (TRP) into indole-containing catabolites that can modulate the immune system in an aryl hydrocarbon receptor (AHR)-dependent manner, contributing to intestinal and systemic homeostasis (Supplementary Table 2).

Humans obtain TRP, which is an essential aromatic amino acid, mainly from a protein-rich diet (including eggs, tuna fish, meat, cheese, beans, and nuts).^134^ In the gastrointestinal tract, TRP is catabolized mainly through three pathways.^135–138^ Initially, more than 90% of dietary TRP is metabolized into kynurenine (Kyn) in immune cells and epithelial cells in an enzyme indoleamine 2,3-dioxygenase 1 (IDO1)-dependent manner.^135,136^ Then, specific intestinal flora convert TRP into indole and indole derivatives as endogenic physiological AHR agonists.^137^ Ultimately, TRP metabolites such as serotonin (5-hydroxytryptamine [5-HT]) are generated in enterochromaffin cells through TRP hydroxylase 1.^138^

### TRP metabolites suppress intestinal inflammation and tumorigenesis

**Fig. 3:**
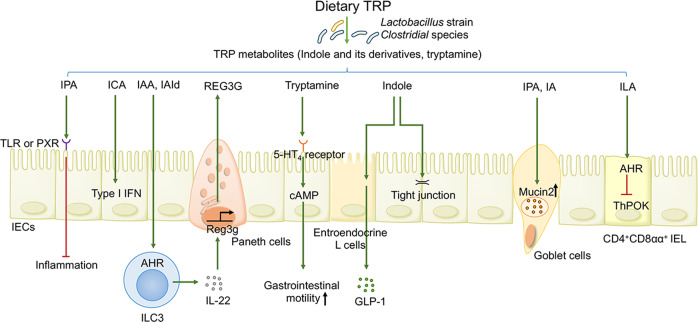
Bacterial catabolism of TRP impacts the host–microbiome interface and immune and metabolic functions. Indoles and their derivatives facilitate the release of AMP and mucin by Paneth cells and goblet cells, respectively, which helps to fortify intestinal barrier integrity. Tryptamine accelerates gastrointestinal motion by acting on the serotonin receptor on IECs. Indole also stimulates enteroendocrine L cells to produce GLP-1, thus maintaining glycometabolism homeostasis. *Lactobacillus reuteri*-derived ILA drives the development of CD4^+^CD8αα^+^ IELs to prevent colitis

First, it is well recognized that microbially TRP metabolites, namely, indole and its multifarious derivatives, play central roles in ameliorating intestinal inflammation and conferring protection against carcinogenesis in an AHR-dependent manner. Diminished expression of AHR in intestinal tissues has been revealed in patients with IBD.^135^ Studies using AHR-deficient mice have also revealed that impaired AHR signaling correlates with diminished levels of IL-22-producing ILC3s and consequently culminates in a worsening of colitis.^139^ Interestingly, alpinetin (an AHR agonist)-mediated AHR activation modulates miR-302/DNMT-1/CREB signaling, thereby increasing Treg differentiation and conferring protection against colitis.^140^ Additionally, IEC-specific AHR deletion contributes to dysfunctional Wnt-β-catenin signaling, which largely impairs the differentiation of ISCs into IECs due to unrestricted ISC proliferation, rendering mice susceptible to inflammation-induced colonic tumorigenesis.^141^ This phenotype could subsequently be rescued via treatment with AHR agonists.^141^ These results highlight the importance of AHR in intestinal homeostasis. Studies using CARD9 (caspase recruitment domain-containing protein 9, an IBD susceptibility gene)-knockout mice have also displayed an impaired immune response to pathogen *Citrobacter*, along with decreased levels of indole-3-acetic acid (IAA) and insufficient IL-22 production by ILCs.^135^ Further deciphering the mechanisms, the enhanced susceptibility of CARD9^−/−^ mice to IBD is primarily attributed to the inability of their microbiome to convert TRP into AHR agonists, indicating that CARD9 affects the dynamic composition of the gut microbiome as well as TRP metabolism.^135^ In contrast, this adverse phenotype can be counterbalanced by colonization with *Lactobacillus* strains that can metabolize TRP into AHR agonists or by supplementing the diets of mice with AHR ligands.^135^ Intriguingly, overexpression of cytochrome p450 family proteins such as Cyp1A1 in mice stimulates the depletion and inactivation of natural AHR ligands, exhibiting decreased levels of AHR-dependent ILC3s and Th17 cells, as well as a failure to withstand enteric infection, which can be reversed by dietary AHR ligands.^142^ Some studies underscore the functional immunoregulatory capabilities of certain microbially TRP metabolites. For example, the gut commensal *L. reuteri* facilitates the catabolism of dietary TRP into AHR agonist indole-3-lactic acid (ILA). The subsequent AHR signaling in CD4^+^ intraepithelial lymphocytes (IELs) lowers the expression of transcription factor T helper-inducing POZ/Krüppel-like factor (also known as ZBTB7B) and further triggers RUNX3 expression, thereby contributing to the development of immunoregulatory T cells (CD4^+^ CD8αα double-positive IELs) to prevent intestinal inflammation (Fig. 3).^143^ Thus, the intricate and dynamic TRP metabolite-AHR crosstalk can modulate intestinal immunity through parallel mechanisms. Independent of AHR, microbial-derived indole-3-carboxaldehydecan elicit a protective type I IFN-dependent signaling response in IECs to protect against intestinal inflammation resulting from myeloablative chemoradiation and acute GvHD.^144^

### Microbially derived indole derivatives modulate intestinal barrier function

Furthermore, gut microbial-derived indole derivatives are also endowed with additional far-reaching functions, including strengthening the intestinal barrier integrity and conferring resistance against enteric pathogens (Fig. 3). *Lactobacillus reuteri* metabolizes TRP into indole-3-aldehyde (IAId) that activates IL-22-producing ILC3s in an AHR-dependent manner, thereby exerting colonization resistance against mucosal *Candidiasis*.^137^ Mdo-miRNA7267-3p, a newly identified plant-derived exosomal microRNA, boosts AHR-dependent production of IL-22 by ILC3s by accelerating indole-3-carbaldehyde (also known as indole-3-aldehyde, I3A) production, ultimately strengthening barrier function.^145^ Additionally, independent of AHR, indole facilitates the expression of apical junction proteins involved in the maintenance of IEC structure and function.^134^ In addition, indole participates in reinforcing colonization resistance against enteric pathogens by downregulating the expression of their virulence repertoire^146^ and mitigating their invasiveness.^147^ *Peptostreptococcus russellii*, a novel commensal bacteria, harbors gene clusters involved in converting TRP into indoleacrylic acid (IA), which enhances the differentiation and expression of goblet cell-associated genes such as *Muc2*, ultimately leading to decreased susceptibility to intestinal injury.^148,149^ A similar mechanism has been demonstrated for indole-3-propionic acid (IPA) in mice fed a HFD.^150^ IPA also diminishes intestinal mucosal permeability and suppresses inflammation in a pregnane X receptor- and TLR4-dependent manner.^151^ Both in vitro and in vivo experiments have shown that IPA inhibits the growth of *Mycobacterium tuberculosis* and decreases their intracellular TRP level.^152^ Gut symbiont *Clostridium sporogenes* (*C. sporogenes*) produce IPA in mice following intestinal colonization.^13^ Notably, an intact *fldC* gene is essential for *C. sporogenes*-mediated IPA generation. Mice colonized with fldC mutant *C. sporogenes* fail to generate IPA and exhibit enhanced intestinal permeability and an increased number of circulating myeloid and lymphoid cells.^13^ The results of this study demonstrate the significance and necessity of specific microbial genes in microbial metabolite production and host immunity.

Aberrant alterations including active IDO1, increased levels of Kyn and diminished IAA are observed both locally in the gut and systemically in patients with IBD, potentially reflecting metabolic reprogramming from microbial to host-dominant metabolism under pathological conditions.^135,136^ Notably, IDO1-mediated generation of the endogenous AHR ligand derived from TRP potentially induces intestinal inflammation.^153,154^ Mechanistically, a class of oxazole-containing compounds derived from the diet, environment and gut microbiome boosts the formation of TRP-derived metabolites (such as Kyn) in response to activated IDO1 and consequently activates AHR in IECs, which suppresses the CD1d-mediated generation of IL-10 by IECs and induces IL-13- and IFN-γ-producing invariant natural killer T (iNKT) cell-mediated inflammation in the gut.^153^ Collectively, these findings advance our understanding of the molecular mechanism underlying the role of environmental oxazolone-like molecules in intestinal immunity. Questions regarding what alternative or redundant intestinal immune cells respond to the IDO1-AHR axis and the functional modulation of IDO1-mediated responses in additional animal models remain to be investigated.^155^

### The immunoregulatory effects of TRP metabolites on extraintestinal organs

**Fig. 4:**
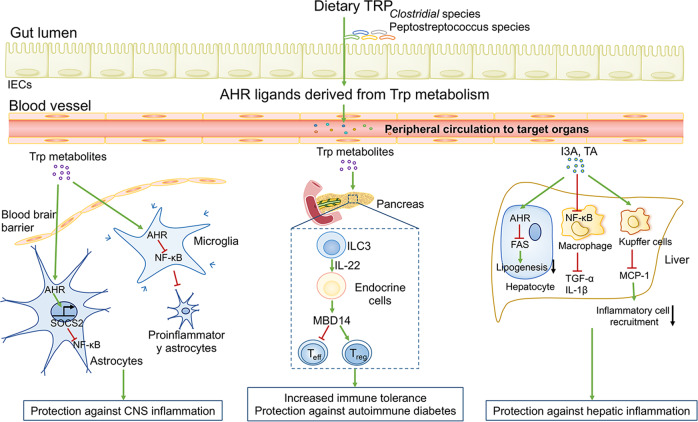
Effects of gut microbiome-derived TRP metabolites on distant organs. Microbially derived metabolites can systemically influence remote tissues, such as the brain, pancreas, and liver. Microbial tryptophan TRP metabolites suppress the proinflammatory activity of astrocytes to inhibit CNS inflammation. TRP metabolites also increase Treg cells while decreasing effector T cells to prevent autoimmunity diabetes. I3A elicits overall instrumental immune effects to inhibit hepatic inflammation via diminishing the generation of proinflammatory cytokines (such as TGF-α, IL-1β, and MCP-1)

The microbially derived TRP metabolites can enter the circulatory system and thus exert immunoregulatory effects on anatomically remote organs, including the brain, liver, and pancreas (Fig. 4). In pediatric patients with multiple sclerosis (MS), the relative abundance of TRP and indole lactate are negatively associated with the risk of MS.^156^ In particular, a protective role of TRP metabolite components in CNS inflammation has been observed in an animal model of experimental autoimmune encephalomyelitis (EAE) that can replicate many of the characteristics of MS. TRP deficiency or specific deletion of AHR in astrocytes drives the recruitment of peripheral inflammatory cells to the brain and further potentiates the pathogenic and neurotoxic activities of astrocytes, thus amplifying local inflammation.^157,158^ Microbially derived TRP metabolites can cross the BBB and further regulate the immune activity of microglia and astrocytes through AHR-driven mechanisms.^157,159^ Gut microbiome-derived TRP metabolites such as indole-3-sulfate, IPA, and IAId signal through AHR in astrocytes to negatively modulate nuclear factor-κB (NF-κB) activation through the suppressor of cytokine signaling 2 (SOCS2), thereby mitigating CNS autoimmunity.^158,160^ Moreover, AHR interacts with the genes that encode the proteins vascular endothelial growth factor B (VEGF-B) and TGF-α, suppressing the transcription of the former while potentiating that of the latter. In this manner, AHR activation in microglia dampens the responsiveness of neighboring astrocytes to CNS inflammation.^157,159^

Furthermore, microbially derived AHR agonists also exert an effect on the pancreatic immune cells involved in autoimmune diabetes progression. Mechanistically, microbial-derived I3A elicits IL-22 production by pancreatic ILCs. ILC-derived IL-22 further promotes pancreatic endocrine cells to express mouse β-defensin 14, which supports the development of pancreatic immunosuppressive macrophages and Treg cells through activation of TLR2 on IL-4-secreting B cells and thus prevents autoimmune diabetes.^128^

The suppressive effect of microbially produced indole derivatives on the hepatic inflammatory response has drawn much attention recently. For example, indole suppresses NF-κB activation while activating NLRP3 signaling in Kupffer cells to counteract LPS-induced hepatic inflammation in mice.^161^ IAA directly diminishes fatty acid- and LPS-induced proinflammatory cytokine (such as TNF-α and IL-1β) generation in macrophages by suppressing the NF-κB pathway and abrogating the recruitment of cells to chemokines.^162^ In a mouse model with alcoholic liver disease (ALD), administration of IAA boosts AHR-dependent IL-22 production by ILC3s and further maintains the expression of regenerating islet-derived protein III-gamma (REG3G, C-type lectin involved in epithelial barrier integrity), thereby dampening the translocation of bacteria to the liver as well as ALD progression.^163^ Collectively, these encouraging findings describe the long-distance regulation of immune cells in extraintestinal organs by gut microbiome-derived TRP metabolite signaling through AHR.

### TRP metabolite-mediated modulation of host metabolism

Gut microbial TRP metabolites participate in the modulation of anorectic hormone generation and glucose and insulin-associated metabolism. Both patients and animal models with metabolic syndrome display gut microbiome deficiencies in converting TRP into indole and its derivatives as AHR agonists.^164,165^ Administration of AHR agonists or a *Lactobacillus* strain that naturally generates AHR ligands restores AHR signaling to increase GLP-1 secretion, ultimately ameliorating metabolic syndrome.^164^ In addition, intestinal IDO1 deletion or inhibition improves insulin sensitivity in obese mice, which is largely attributable to the rewiring of TRP metabolism from Kyn production towards a microbiome-dependent production of indole derivatives and IL-22, providing support for the notion that gut microbiome-derived AHR agonists are responsible for shaping metabolic homeostasis.^154^ Indeed, colonic enteroendocrine L cells increase GLP-1 secretion following a short exposure to physiological levels of indole, but this phenotype is suppressed during prolonged exposure.^166^ TRP-derived indole generated by the gut microbiome suppresses the expression of the miR-181 family in white adipocytes in mice to ameliorate HFD-induced obesity and insulin resistance, highlighting a novel mechanism in the gut–fat axis.^165^ By acting through AHR in hepatocytes, I3A attenuates cytokine-induced lipogenesis, offering promise for gut microbially derived TRP metabolites to treat metabolic disease by targeting lipid metabolism.^162^

### TRP metabolite-mediated modulation of host neurotransmitters

It has been established that certain bacteria (such as *Clostridial* species) possess TRP decarboxylase, which converts TRP into the neurotransmitter tryptamine.^167^ The microbiome-derived tryptamine serves as a ligand for the gut epithelium-expressed serotonin receptor 4 (also known as 5-HT receptor 4, 5-HT_4_ receptor), contributing to heightened intracellular cAMP levels and increased fluid secretion into the gut to accelerate gastrointestinal motility,^168,169^ indicating therapeutic potential in gastrointestinal motility disorders such as irritable bowel syndrome (IBS).

As GPCRs interact intimately and mutualistically with a myriad of microbiota and their metabolites and perform essential physiological functions,^170^ high-throughput activity-based screening using potential host GPCRs is effective to narrow down complex metabolite libraries and identify responsible effector gut bacteria and their corresponding bioactive microbial metabolites that are capable of activating both well-characterized and orphan GPCRs.^171^ For example, *Morganella morganii* (*M. morganii*), a newly identified gut bacterium, produces histamine from dietary histidine to potentiate gastrointestinal motility. This beneficial effect is similar to that of TRP-derived tryptamine.^171^ *Morganella morganii* can convert l-phenylalanine (L-Phe) into the potent psychoactive trace amine phenethylamine, which crosses the BBB and drives lethal phenethylamine poisoning in combination with monoamine oxidase inhibitor administration.^171^

Collectively, similar to SCFAs, TRP metabolites generated by gut commensals are commonly endowed with the capacity to stimulate anti-inflammatory pathways, maintain intestinal barrier integrity, and ameliorate metabolic disorders. Certain metabolites, such as 5-HT and tryptamine, function as neurotransmitters to effectively modulate the gut–brain axis.

### Secondary bile acids

The liver metabolizes cholesterol into primary bile acids that are retained in and released from the gallbladder into the small intestine where they can be utilized to dissolve dietary lipids and fat-soluble vitamins. The large proportion of primary bile acids are assimilated in the ileum and recycled back to the liver; a small proportion (~3%) enter the large intestine in which they are easily deconjugated and thus converted by the gut bacteria into secondary bile acids that exert pleiotropic effects on host immunity.^172^

### Secondary bile acids modulate gut barrier function

One of the essential immunoregulatory functions of secondary bile acids is to augment gut barrier function through multiple mechanisms, including maintaining intestinal barrier integrity and inhibiting pathogen colonization. As an example, deoxycholic acid (DCA), one of the secondary bile acids, downregulates prostaglandin E2 (PGE2) synthesis in a farnesoid X receptor (FXR)-dependent manner, thereby accelerating intestinal crypt regeneration and wound repair.^173^ Supplementation with a mixture of lithocholic acid (LCA, another secondary bile acid) and ursodeoxycholic acid (UDCA) is favorable for maintaining gut barrier integrity through activation of the FXR-FGF15 pathway.^174^ Additionally, several studies have revealed that specific bacterial-derived secondary bile acids favor an intestinal microenvironment that is detrimental for pathogen colonization. The archetypal example is *Clostridium scindens* (*C. scindens*), which harbors a beneficial metabolic function enabling the 7α-dehydroxylation of primary bile acids into DCA, conferring resistance to pathogen *C. difficile* expansion. Antibiotic-mediated disruption of specific microbiota such as *C. scindens* leads to the accumulation of primary bile acids, thereby increasing spore germination of *C. difficile*.^175,176^ Consistently, a recent study provides correlative support for the notion that DCA reduces pathogen burden, and it has been shown that oral administration of DCA is responsible for inhibiting *Campylobacter jejuni*-induced colitis in mice by suppressing the expression of proinflammatory genes in IECs.^177^ In addition, both DCA and LCA are similarly shown to decolonize pathogen *C. difficile*, albeit by a distinct mechanism that DCA and LCA potentiate the activity of TRP-derived antibiotics secreted by the known DCA and LCA producers *C. scindens* and *C. sordellii*.^178^ Notably, whether microbiota-derived LCA exerts a beneficial or harmful part in pathogen intestinal colonization remains elusive. Indeed, it was recently reported that LCA is conducive to certain biological processes in Vancomycin-resistant *Enterococcus* (VRE), including the formation of long chains and increased biofilm formation, thereby promoting the expansion of VRE.^179^ These studies provide pivotal information regarding how secondary bile acids may signal to other pathogens and host cells, which offers promise for the rational design of clinical translation based on microbiota-derived metabolites. In fact, oral administration of UDCA is therapeutically efficacious in a case of patient with *C. difficile* infection (CDI)-associated pouchitis.^180^ However, the utilization of live *C. scindens* appears to offer advantages over exogenous administration of metabolites, as *C. scindens* exerts a two-pronged approach to suppress pathogens, namely, enhancing secondary bile acids while simultaneously diminishing primary bile acids.^61^

### Secondary bile acids modulate tumorigenesis

Despite the beneficial effects on gut barrier function, the carcinogenic effects of secondary bile acids and the underlying mechanisms have become the focus of microbial metabolomic analyses. A meta-analysis integrated with eight studies of colorectal cancer (CRC) patients receiving fecal metagenomic sequencing revealed the increased generation of secondary bile acids from CRC metagenomes.^181^ Similarly, DCA is significantly elevated in patients with multiple polypoid adenomas and/or intramucosal carcinomas.^182^ A HFD induces a remarkable increase in DCA, and tauro-β-muricholic acid triggers aberrant proliferation and DNA damage in Lgr5-expressing (Lgr5^+^) cancer stem cells by inhibiting intestinal FXR, thereby accelerating CRC progression.^183^ Furthermore, the onset and development of primary HCC is also modulated by secondary bile acids through multiple distinct mechanisms, such as DNA damage, inflammation-associated tumorigenesis, hepatotoxicity,^184,185^ and favoring an immunosuppressive tumor microenvironment by diminishing the accumulation of hepatic NKT cells.^186–188^ Thus, secondary bile acids show distinct phenotypic effects on the host, including triggering tumorigenesis while maintaining intestinal barrier function.

### Microbially derived succinate

**Fig. 5:**
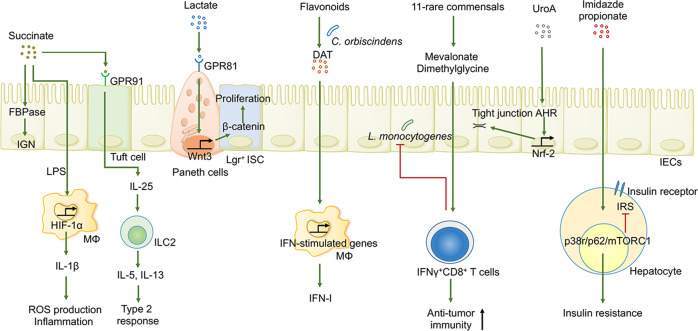
Additional gut microbial metabolites regulate host immunity and metabolism. Microbiome-derived succinate can facilitate IGN and upgrade type 2 immunity against parasitic infection. Lactate participates in intestinal wound repair by triggering ISCs in a GPR81-dependent manner. DAT confers resistance against virus infection by amplifying IFN-I signaling. A mixture of 11 rare, commensal-derived bacteria facilitates the development and accumulation of IFNγ^+^CD8^+^ T cells to enhance anti-tumor immunity and anti-intracellular pathogen infection. UroA can modulate junction proteins, which directly regulate epithelial permeability. Imidazole propionate obstructs the insulin receptor signaling pathway and results in the onset of insulin resistance following the inhibition of IRS function

There is growing awareness that, in addition to SCFAs, microbial fermentation of dietary fiber (especially polysaccharides and oligosaccharides) can produce considerable levels of succinate, which is classically considered the precursor of propionate in microbial metabolism and an intermediate in tricarboxylic acid cycle as well as a crucial ligand for GPR91 (also known as SUCNR1)^14^ (Fig. 5).

### Succinate-mediated modulation of host metabolism

Microbially generated succinate is increasingly recognized to benefit the host as an orchestrator of metabolic homeostasis. For example, dipeptidyl peptidase-4 inhibitors (DPP-4i), a commonly used antidiabetic agent, drive significant enrichment of Bacteroidetes and induce a functional shift in the intestinal microbial metabolome, particularly enhancing succinate generation. In this manner, the treatment of HFD mice with DPP-4i improves glucose homeostasis.^189^ Analogous to the properties of propionate, succinate is sufficient to activate IGN in obese mice by enhancing the activity of fructose-1,6-bisphosphatase (the rate-limiting enzyme in IGN), thereby leading to the amelioration of insulin sensitivity and glucose tolerance and the modulation of body weight.^174,190^ In addition, elevated circulating levels of succinate in mice swiftly drive thermogenic respiration in brown adipose tissue, a function that protects against diet-induced obesity and hyperglycemia by initiating UCP1-dependent thermogenesis and supporting the generation of reactive oxygen species (ROS) induced by succinate dehydrogenase (SDH)-mediated oxidation.^191,192^

Notably, inconsistent with the above point of view, data obtained from human prospective studies revealed that obesity and impaired glucose homeostasis correlate with increased systemic concentrations of succinate concomitant with the elevated levels of succinate-producing microbiome and diminished levels of succinate-consuming bacteria.^193^ Therefore, whether succinate potentially acts as a detrimental metabolic product derived from microbiota in humans or whether disrupted gut microbiome and aberrant intestinal permeability under obesity conditions may contribute to elevated succinate are topics that require further investigation.

### Succinate-mediated immunomodulation

Succinate has recently attracted great attention because of its role in immune modulation. As an example, succinate in mice elevated by the administration of longan polysaccharide may boost host immune function in the context of stress, which can be attributed to favorable alterations in the intestinal immunity index such as IgA, IL-6, IFN-γ, and TGF-β.^194^ Similarly, the elevated succinate levels resulting from polyphenols in combination with HFD exert inhibitory effects on the growth and proliferation of colon cancer cells as well as angiogenesis.^195^

In 2018, experimental studies revealed the essential nature of succinate; it is dependent on GPR91 to mediate a robust type 2 innate immune response against luminal protozoa and helminths, which is required for the efficient expulsion of pathogens and the host’s return to homeostasis.^196–198^ More precisely, GPR91-expressing tuft cells are the dominant source of IL-25 in the gut by perceiving and recognizing succinate derived from protists and helminths. Elevated IL-25 is responsible for eliciting ILC2 cells to generate IL-13 and IL-5, which in turn facilitate the proliferation of tuft cells and goblet cells as well as the prevention of anti-parasitic infection.^196–200^ Notably, the perception of tuft cells to protist-derived succinate is dependent on GPR91, but helminths potentially activate tuft cells in an GPR91-independent manner.^200^ In addition, both FFAR3 (a receptor of SCFAs) and GPR91 are expressed on tuft cells, and only succinate can directly modulate the tuft cell-ILC2 circuit.^77^

However, data from patients with Crohn’s disease (CD) have revealed that increased succinate in serum and the intestine as well as elevated expression of its receptor SUCNR1 in the gut participate in the deterioration of intestinal inflammation and fibrosis in CD patients.^201^ LPS induces remarkably elevated levels of succinate, which can exacerbate inflammation. Succinate fortifies the transcription factor HIF-1α, supporting the production of the proinflammatory cytokine IL-1β by macrophages.^202^ Moreover, SDH-mediated mitochondrial oxidation of succinate triggers a metabolic reorientation that shifts mitochondria away from ATP synthesis and towards ROS generation, thereby supporting a proinflammatory state.^203^ Further comprehensive studies are needed to identify the functional regulatory effects of succinate on intestinal immunity and inflammation.

### Lactate-mediated immunomodulation

Lactic acid (lactate), another microbial metabolite derived from the diet and commonly found in milk, harbors a wide array of metabolic and immune properties, including serving as HDAC inhibitors, an essential energy source for cell renewal, and signaling molecules^14^ (Fig. 5).

Oral administration of lactate derived from *L. helveticus* elicits the dendrite extension of gut CX3CR1^+^ cells into the intestinal lumen to bind antigens in a GPR31-dependent manner, triggering an intensive antigen-specific immune response against *Salmonella* infection.^204^ Similarly, the administration of *L. lactis* to infected neonatal mice effectively diminishes the infectious burden of intestinal pathogen *Vibrio cholerae* and consequently enhances survival, which is largely attributed to the local abundance of lactic acid.^205^ These results have revealed that lactate-mediated immunomodulation in the gut can provide support for decolonizing intestinal pathogens. In addition, the increased lactate levels owing to supplementation with lactate-producing bacteria-type symbionts such as *Bifidobacterium* and *Lactobacillus* stimulates ISC-mediated epithelial development by Wnt/β-catenin signals in Paneth cells and intestinal stromal cells.^206^ Notably, mice with deletion of GPR81 display impaired ISC-mediated epithelial development, indicating that lactate favorably orchestrates the gut barrier function in a GPR81-dependent manner.^206^ In contrast, in *Drosophila* with a null mutation in Peptidoglycan recognition protein-SD, excessive lactate generated from overgrown *L. plantarumin* activates the intestinal NADPH oxidase Nox and thus boosts ROS production, ultimately contributing to intestinal injury and intestinal dysplasia associated with aging.^207^ This example highlights that the beneficial or detrimental effects of lactate are potentially dependent on the dose, experimental model, and host immunocompetence.

### Additional microbially derived metabolites

The above-mentioned metabolites are not all-encompassing; however, high-resolution mass spectrometry and metabolomics are advancing rapidly, which confers an additional lens through which other bacterial-derived metabolites involved in a myriad of diseases can be identified (Fig. 5).^208^

### Additional metabolite-mediated immunomodulation

One of the most pronounced examples is desaminotyrosine (DAT), which is a bacterial metabolite derived from flavonoids and produced by the gut commensal *C. orbiscindens*. This metabolite is sufficient to boost type I IFN production by amplifying IFNAR and STAT1 signaling, thereby conferring protection against influenza infection.^209^ Treating BM-derived macrophages with DAT triggers IFN-stimulated gene transcription and diminishes viral RNA levels in these cells following poliovirus as well as oral reovirus infection.^210,211^ These results highlight the causal role of bacterial-derived metabolites in combating viral infection. Similarly, ascorbate is considered a bioactive microbial metabolite related to CD and can induce T cell apoptosis by targeting the energy metabolism of activated effector CD4^+^ T cells.^212^ In addition, circulating metabolites such as mevalonate and dimethylglycine, which are produced by a consortium of 11 rare bacteria (predominately *Bacteroidetes*) isolated from healthy individuals feces, seem to potentiate the systemic development of IFN-γ^+^ CD8+ T cells, thereby combating the intracellular pathogen *Listeria monocytogenes* (*L. monocytogenes*) and enhancing ICI-mediated anti-tumor immunity in mice with melanoma.^43,213^ This groundbreaking research has demonstrated that rare microbiome members potentially harbor profound effects on host immunity. Another microbial metabolite is urolithin A (UroA), derived from polyphenolics abundant in fruits, which can upregulate epithelial tight junction proteins through activation of AHR-NRF2-dependent pathways, enhancing barrier function and ameliorating intestinal inflammation.^214^ Recently, in a phase I clinical trial, short-term oral supplementation of UroA in elderly people who lack exercise is safe and exerts an instrumental effect on improving skeletal muscle health and decelerating aging, which is largely attributable to the capability of UroA to activate mitochondrial autophagy.^215^

Notably, certain microbial metabolites appear to detrimentally impact host immunity. For example, spermine, putrescine, and histamine suppress NLRP6 inflammasome activation and reduce subsequent IL-18 secretion, thereby impairing gut epithelial barrier integrity.^216^ Similarly, microbiome-derived 1,2-propanediol strengthens virulence factor expression in pathogens, supporting the intestinal colonization and expansion of pathogens such as *C. rodentium*.^217^

### Additional metabolite-mediated modulation of host metabolism

Imidazole propionate, produced by type 2 diabetes-associated bacteria as a metabolite of histidine, is heightened in type 2 diabetes and impairs glucose tolerance and insulin signaling, a process achieved by inhibiting insulin receptor substrate (IRS) in a p38g/p62/mTORC1-dependent manner.^218^ In a similar vein, the gut microbiome that harbors tyrosine phenol-lyase can catabolize dietary tyrosine into the precursor phenyl sulfate. Subsequently, the increased circulating levels of phenyl sulfate exert deleterious effects on the kidneys via damaging podocytes, accelerating glomerular basement membrane thickening and inducing proteinuria.^219^ These results suggest that therapeutically targeting the microbiota responsible for these metabolic pathways can ameliorate symptoms of metabolic disease.

In summary, in addition to common microbial metabolites, a myriad of novel small molecules or catabolites that are generated by the gut microbiome function as chemical messengers to transmit microbial-derived signals to various parts of the host, contributing to the dynamic interaction between the gut microbiome and humans and exerting instrumental or detrimental effects on the outcomes of multiple disorders, such as intestinal inflammation, autoimmune disease, metabolic diseases, and tumors. Further comprehensive studies are warranted to unravel the roles of these additional metabolites and the underlying mechanisms.^220^

### Crosstalk between the intestinal microbiome and host immune system

**Fig. 6:**
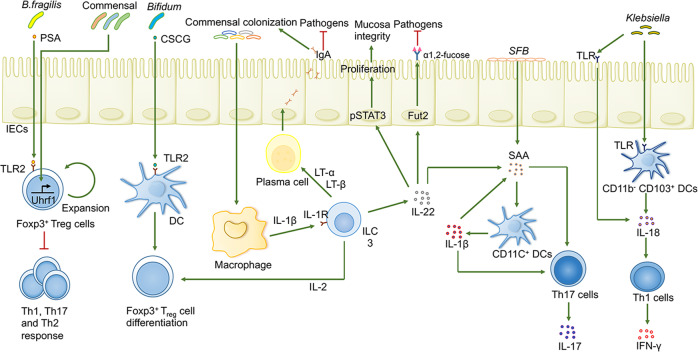
The crosstalk between the gut microbiome and CD4^+^ T cells as well as ILC3s. The specific gut microbiome is sufficient to induce the development of Treg, Th17, and Th1 cells, as well as IgA secretion by plasma B cells. ILC3s play a central role in such an immune network to maintain gut homeostasis through the exclusion of pathogens, maintenance of the intestinal mucosal barrier, and anti-inflammatory effects

In addition to the modulation of microbiome-derived metabolites on host immune and metabolism, the gut microbiome also establishes fine-tuned communications with the host immune system (especially CD4^+^ T cells) through multiple metabolite-independent mechanisms, as described below and depicted in Fig. 6.

### Microbiota-mediated manipulation of Treg cells

CD4^+^Foxp3^+^ Treg cells have been extensively identified as an indispensable component that is responsible for immune tolerance to nonpathogenic antigens as well as exerting suppressive effects on pathogen-induced tissue damage mediated by Teff cells.^40,221–224^ Foxp3^+^ Treg cells are substantially enriched in intestinal lamina propria,^225–227^ and they are composed of thymus-derived Treg cells that impede Teff cell-induced inflammation in the host^222^ and peripherally differentiated Treg (pTreg) cells that confer immunological tolerance to nonpathogenic antigens.^40^ More precisely, Foxp3^+^ Treg cells in the gut can be subclassified into at least three subsets based on their expression of RAR-related orphan receptor γt (RORγt), GATA3, zinc-finger protein Helios, and the receptor neuropilin 1 (Nrp1)^222,223,228,229^ (Table 1).

**Table 1:** Effects of the gut microbiome and diet on subsets of CD4^+^Foxp3^+^ Treg cells in the gut

Subsets	Molecular expression	Inductive sites	Inducer	Function	Refs.
pTreg cells	Primarily express RORγt and CTLA-4, generally lack Helios and Nrp1	Colon	Induced by the gut microbiome	Inhibit the aberrant activation of myeloid, γδ T, and Th17 cells through IL-10	[228, 229]
	Express neither RORγt nor Nrp1, Helios	Small intestine	Induced by dietary antigens, unaffected by the absence of the gut microbiome	Decrease susceptibility to food allergies	[223, 228]
tTreg cells	Mostly express GATA3, ST2, Nrp1, and Helios	Thymus	Induced by self-antigens, unaffected by the absence of the gut microbiome	Mediate immune regulation under conditions of inflammation induced by self-antigens	[222, 228]

The perturbation of gut symbionts is associated with immune dysfunction partially due to impaired Foxp3^+^ Treg cells.^230^ Adaptive immunity is sparsely developed in both GF- and antibiotic-treated mice, which is characterized by a paucity of intestinal Treg cells as well as proneness to Th2-, Th1-, or Th17-mediated autoimmune responses, a phenotype that can be reversed by replenishment of the microbiome, indicating a positive regulatory effect of the gut microbiome on the activation, polarization, and function of Treg cells.^11,228,231^ Generally, mice receiving microbiomes from healthy donors are able to induce more RORγt^+^ Treg cells.^22^ In the context of the normal gut microbiome, CX3CR1^+^ monocyte phagocyte (MNP)-derived IL-10 favors the expansion of Treg cells while suppressing the proliferation of proinflammatory Th1 and Th17 cells, contributing to the inhibitory inflammatory response to nonpathogenic antigens in the gut.^11^ Intriguingly, wild-type (WT) mice colonized by *Helicobacter hepaticus* (*H. hepaticus*), a potentially pathogenic bacterium, support the growth of RORγt^+^FOXP3^+^ iTreg cells that selectively inhibit proinflammatory Th17 cells in a c-Maf-dependent manner.^232,233^ Actually, in the setting of gut homeostasis, pathogens such as *Helicobacter* trigger antigen-specific RORγt^+^ pTreg cells that hamper Th17 cell responses, while under inflammatory conditions, such bacteria are able to stimulate antigen-specific Th17 cells that sense and identify identical epitopes.^232,234^ These reports have conferred a novel lens that such a RORγt^+^ pTreg cell subset is endowed with clinical correlation because of its potential capability to modulate intestinal Th17 cell-type immune responses.^228,234,235^ In addition, impairments in the differentiation and activation of Treg cells are correlated with severe intestinal disorder, allowing expansion of pathogens while inhibiting the growth of beneficial commensal bacteria.^232,233^ Moreover, the disappearance of pTreg cells exerts detrimental impacts on the intestinal bacterial colonization hierarchy.^236^ Mechanistically, under conditions of pTreg cell deficiency, the microbial-induced type 2 immune response stimulates ILC2 cells and Th2 cells to secrete cytokines (such as IL-13 and IL-5). The recognition of and response to these cytokines by goblet cells subsequently produces anti-microbial molecules (such as Ang4) and thus dampens border-dwelling bacteria colonization and impairs the niche of specific border-dwelling bacteria.^231,236^

Considering the therapeutic potential and remarkable role of Tregs in inflammatory diseases, researchers have been showing enormous interest in the molecular identities of the “effector” components responsible for the induced programs. As demonstrated meticulously above, one of the most pivotal mechanisms for Treg cell induction is the stimulation effects of microbial-produced metabolites, namely, SCFAs. Additional mechanisms of epigenetic regulation are also illustrated. That is, colonization of the gut microbiome in GF mice upregulates the expression of the DNA-methylation adaptor Uhrf1 in Treg cells, accelerating the functional maturation and proliferation of Treg cells.^237^ In addition, the differentiation of gut Treg cells is assumed to be delicately controlled by microbial-dependent extracellular structures or their own products. Colonization of mice with nonpathogenic *E.* *coli* expressing ovalbumin (OVA) peptide at the membrane triggers antigen-specific Foxp3^+^ Treg accumulation.^238^ Consistent with this, *Bacteroides fragilis* (*B. fragilis*)-derived polysaccharide A (PSA) activates TLR2 expressed on Foxp3^+^ Treg cells, boosting the immunosuppressive function and growth of Treg cells while suppressing pathological Th17 cell differentiation.^239,240^ DC-derived cytokines, including IL-10 and TGF-β, are significantly increased through PSA-dependent TLR2 activation, further promoting naive CD4^+^ T cell differentiation into IL-10-producing Tregs.^241^ PSA may be absorbed into *B. fragilis*-produced outer membrane vesicles (OMVs), which are handed to DCs through a noncanonical autophagy pathway. Notably, genes associated with IBD, such as autophagy-related protein 16-like 1 (ATG16L1) and nucleotide-binding oligomerization domain-containing protein 2 (NOD2) are required for this OMV-mediated immunomodulatory to elicit such pathway and prevent colitis, indicating that these genes are dependent on intestinal homeostasis.^242,243^ Indeed, selective deletion of ATG16L1 in T cells elicits spontaneous intestinal inflammation that is characterized by impaired Foxp3^+^ Treg cells and an aberrant type 2 response to nonpathogenic antigens as well as decreased AMPs.^244^ Similarly, specific deficiency of ATG16L1 in IECs contributes to the secretion of proinflammatory cytokines and to the apoptosis of IECs, thereby deteriorating chronic colitis.^245,246^ In addition, bacteria-derived Zwitterionic capsular polysaccharides stimulates Treg cell differentiation and IL-10 production in an antigen-presenting cell (APC)-dependent manner.^247^

The recent shift towards detailed mechanism understanding has also identified individual bacterial members or particular communities that directly contribute to Treg cell development in particular pathways. One of the best-characterized instances is that members of the *Clostridia*, especially clusters IV, XIVa, and XVIII, are endowed with a robust capacity to induce intestinal Treg via multiple distinct mechanisms, including SCFA-mediated induction, stimulating TGF-β production in IECs and IL-2 generation in Teff cells^58,225,248,249^ or activating a MyD88/RORγt pathway in naive Treg cells.^27^ Indeed, *Roseburia intestinalis* (*R. intestinalis*), a typical member of *Clostridia*, appears to be of great importance for increasing Treg cells in a colitis mouse model by reinforcing the secretion of thymic stromal lymphopoietin and TGF-β.^12,114^ Attractively, flagellin, an effective modulator and crucial structure of *R. intestinalis*, exerts anti-inflammatory effects via activating p38-STAT1 to induce lncRNA (HIF1A-AS2) expression.^250^ Nevertheless, the pleiotropic effects of *R. intestinalis* on host immunity have been demonstrated, as oral supplementation with *R. intestinalis* elicits anti-human β2-glycoprotein I autoantibodies and autoimmune pathologies in antiphospholipid syndrome-susceptible mice.^251^

In addition, colonization with non-*Clostridium* strains also orchestrates the CD4^+^ T cell compartment by eliciting programs for intestinal Treg cell accumulation. The above-discussed *B. fragilis* is a classic example. Notably, the protective effect of nontoxigenic *B. fragilis* on colitis is partly in a PSA-independent manner by which the bacterial sphingolipid source limits the expansion of colonic iNKT cells.^252,253^ The recolonization of *Lactobacillus* has also been associated with restoring the proportion of Treg cells and activating DCs in broad-spectrum antibiotic-treated mice.^254^ Cell surface β-glucan/galactan polysaccharides of *B. bifidum* strain are also assumed to be pivotal ingredients responsible for Treg induction and eliciting anti-inflammatory responses through activating TLR2 on DCs.^255^ *A. muciniphila*, considered a potential probiotic, is also endowed with the specific capacity to facilitate Treg differentiation as well as an increase in SCFA generation.^256^

Altogether, these results emphasize the significance of Treg cells in enabling the development of a harmonious coexistence among the host, the trillions of noninvasive, symbiotic microorganisms that comprise the microbiome, and normal dietary antigens. There is considerable overlap in the responses of Treg cells to *Clostridium*, *Lactobacillus*, and *Bacteroides*, suggesting that dynamic interactions between these promising, unconventional probiotics and Treg cells also hold promise for the utilization of various beneficial commensals to target immune system deficits in patients.

### Microbiome-mediated modulation of Th17 cells

RORγt^+^ Th17 cells, a subpopulation of CD4^+^ Teff cells, account for 30–40% of the T cells in intestinal lamina propria and are induced in response to TGF-β and IL-6 or IL-21.^257^ The physiological condition of the Th17 cells is largely dependent on the surrounding cytokine environment. For example, Th17 cells that are differentiated in a setting that includes TGF-β and IL-6 confer protection against extracellular pathogenic infection and support the intestinal barrier integrity in certain scenarios.^155^ Commensal-specific Th17 cells also secrete type 2 cytokines such as IL-5 and IL-13 to aid in repairing acute injury in the mucosa.^258^

In contrast, Th17 cells are also endowed with pathogenic activity following exposure to IL-23 and IL-1β. Pathogen-elicited inflammatory Th17 cells show an intense propensity towards the peripheral release of proinflammatory cytokines and are metabolically skewed towards oxidative phosphorylation, analogously to inflammatory effector cells.^259^ GF mice receiving microbiomes isolated from IBD donors display an enhanced accumulation of pathogenic Th17 cells in the gut, increasing the susceptibility to colitis.^22^ Colitis-induced Th17 cells and the resulting IL-17 secretion also enhances the risk for CDI.^260^ Moreover, gut microbial-induced Th17 cells potentially elicit neurodevelopmental abnormalities in the offspring of pregnant mice in an IL-17A-dependent manner.^261^ In mice with multiple myeloma (MM), *Prevotella heparinolytica* upgrades the differentiation of intestinal Th17 cells and their migration to the BM. The correspondingly enhancive IL-17 triggers STAT3 phosphorylation in murine PCs and activates eosinophils, thereby accelerating MM progression, indicating the far-reaching influence of pathological Th17 cells and IL-17 on hematopoietic malignancy.^262^ Notably, the mucocutaneous pathobiont *Candida albicans* (*C. albicans*) has been described to act as a dominant and direct inducer of human anti-fungal Th17 cells.^263^ Colonization of *C. albicans* in the gut elicits robust proliferation of systemic fungal-specific Th17 cells and IL-17 responsiveness through the circulation of neutrophils through the bloodstream, facilitating protection of the mucosa against pathogens while increasing the susceptibility to allergic airway inflammation due to the inability of *C.* *albicans* to withstand intracellular influenza virus infection.^264^ That study provided novel evidence to indicate that microorganism-induced Th17 cells function as a double-edged sword in immune response, including their pathogenicity in multiple inflammatory diseases while maintaining intestinal barrier function in a noninflammatory manner.^259^ Questions regarding what additional explicit stimuli participate in the development of homeostatic or pathogenic Th17 cells and the mechanisms involved in this process need to be further investigated.

Th17 cells are virtually absent in both GF mice and antibiotic-treated mice.^257^ *Segmented filamentous bacteria* (*SFB*) primarily reside in the terminal ileum and are considered as one of *Clostridium* symbionts and prototypical inducers of intestinal Th17 cells.^61,265,266^ Colonization of *SFB* in mice shows higher levels of ATP that stimulates the secretion of Th17-prone molecules, including IL-6, IL-23, and TGF-β by CD70 (high) and CD11c (low) cells in the lamina propria, leading to a remarkable increase in Th17 cells.^267^ Further describing the underlying mechanisms associated with Th17 cell differentiation, IEC-secreted serum amyloid A (SAA) is of the essence for Th17 cell polarization.^268^ Specifically, *SFB* signals through STAT3 in IECs to stimulate SAA secretion and thereby Th17 cell differentiation.^268^ The tight physical adhesion of *SFB* with the ileal epithelium is sufficient to induce SAA1 by facilitating actin reorganization and upregulating the transcription factor C/EBPδ.^269^ SAA elicits the lamina propria CD11c^+^ DCs to produce IL-1β and IL-23, which synergistically promote IL-22 production in ILC3 cells. ILC3-derived IL-22 further potentiates SAA-mediated IL-1β generation by CD11c^+^ myeloid cells, facilitating the development of RORγt^+^IL-17^+^Th17 cells.^265,266,268,269^ A study using electron tomography revealed another *SFB*-specific pathway and molecule that triggers Th17 cell development. More precisely, adhesion-directed endocytosis transfers the *SFB* cell wall-associated proteins into the cytosol of IECs through a cell division control protein 42 homolog-dependent mechanism. Importantly, *SFB* cell wall-associated proteins are sufficient to elicit Th17 cell development. This *SFB*-specific manner to induce TH17 cells has revealed another intricate crosstalk circuit between IECs and TH17 cells.^270^

The immunological effects of *SFB*-induced Th17 cells are context-dependent. *SFB*-mediated Th17 cell differentiation can combat infection associated with pathogens, including *C. rodentium*,^265^ *Salmonella enterica*, and *Klebsiella pneumoniae*^61^ while worsening autoimmune diseases such as EAE^271,272^ and autoimmune arthritis.^273^ Nevertheless, whether *SFB*-induced beneficial Th17 cells are sufficient to combat enteropathogenic infection is still controversial. Despite participating in maintaining intestinal homeostatic balance, *SFB*-induced homeostatic Th17 cells fail to secrete IFN-γ following stimulation and could not eliminate *C. rodentium*-elicited infection.^259^ Notably, *SFB* also exert impacts on numerous additional aspects of the immune system by facilitating the development of intestinal lymphoid tissue and mediating IgA responses.^274^ Consequently, *SFB* potentially influences multiple immunomodulatory responses via a common pathway. Mutually, Th17 cells control the colonization level of *SFB* in the intestine through IL-17R-mediated expression of α-defensins, Nox1, and Pigr. The aberrant IL-17–IL-17R axis results in gut dysbiosis, allowing the expansion of intestinal pathogens and increasing the risk of autoimmune inflammation.^275^

The gut microbiome with antigenic specificity is one of the momentous determinants for the type and function of induced Teff cells, consistent with the notion that the *SFB* antigen expressed by *L. monocytogenes* can be regarded as an effective inducer to trigger IFN-γ-producing Th1 cells.^276^ Additional symbiotic bacterial species can also serve as inducers of intestinal Th17 cell development in *SFB*-independent manners. For instance, *B. adolescentis*, a symbiotic bacterial species derived from humans, and a mixture of 20 strains derived from ulcerative colitis (UC) patients induces intestinal Th17 cells in murine, a process intimately correlated with the ileal epithelium adhesion.^269,277^ In addition, commensal *Propionibacterium* strain UF1 (*P.* *UF1*), a potential beneficial strain isolated from premature infants, significantly induces protective bacteria-specific Th17 cells and shapes favorable immune profiles locally and systemically to protect against *L. monocytogenes* infection in neonatal mice.^277^ A similar study using mice colonized by *P. UF1* facilitates Th17 cell differentiation dependent on the bacterial surface layer (S layer) of dihydrolipoamide acetyltransferase, a prime protein expressed on the S layer of *P.* *UF1*. Moreover, *P. UF1* binds to SIGNR1 on DC to regulate intestinal phagocytic cells, thereby conferring protection against pathogen infection,^278^ highlighting the potential benefit of *P. UF1*-mediated immunomodulation in targeted infectious enteropathy. In contrast, in experimental models, depletion of *L. murinus* induced by a high salt diet triggers intestinal Th17 cell dysregulation and consequently results in autoimmune disorders as well as hypertension, which can be rescued by supplementation with *L. murinus*.^279,280^ Moreover, diet containing high salt enhances the level of intestinal Th17 cells and plasma IL-17, which leads to endothelial dysfunction and cognitive impairment by decreasing NO production in cerebral endothelial cells, giving rise to the novel connection between the diet and the gut–brain axis.^281^

Hence, in accordance with the existing reports, epithelial adhesion is pivotal in the process of bacteria-induced Th17 cells. The question of which specific microorganisms are sufficient to stimulate the development of potentially pathogenic or homeostatic Th17 cells still needs to be thoroughly investigated. How to differentiate the Th17 cells that facilitate gut barrier function from those that are implicated in pathogenic proinflammatory responses is another unanswered question.

### Microbiota-mediated modulation of Th1 cells

The capability of the gut microbiome to manipulate the induction of Th1 cells has also drawn much attention recently. For instance, *Klebsiella* species, which usually reside in the oral cavity, can ectopically colonize the gut and preferentially elicit a proinflammatory Th1 response via either IECs or DCs.^282^ TLR and IL-18 signaling from IECs to the CD11b^−^ CD103^+^ DC subset contributes to *Klebsiella*-mediated Th1 cell induction and increased expression of IFN-γ.^282^ In addition, microbial-derived products also participate in modulating proinflammatory Th1 cell induction. *Bilophila wadsworthia* (*B. wadsworthia*) has the capacity to convert deconjugated taurine into sulfite, which is utilized for its energy metabolism. The subsequent proliferation of *B. wadsworthia* triggers a proinflammatory IFN-γ-producing Th1 cell response and exacerbates colitis in IL-10^−/−^ mice.^283^ Intriguingly, as a pathway of self-modulation, Th1 cells can also generate IL-10 to convert proinflammatory Th1 cells into T cells with regulatory activity,^284^ and signals from the microbiome-derived SCFAs potentially manipulate such a Th1 response, which may confer unique opportunities for therapeutic intervention in Th1-driven immune diseases.^64^

### The gut microbiome and IgA

IgA is the most abundant secretory immunoglobulin isotype at mucosal surfaces. Polymeric IgA interacts with the polymeric immunoglobulin receptor (pIgR) on the basolateral surface of the gut epithelium and is further transported to the apical surface in a transcytosis-mediated manner. IgA is secreted into the intestinal lumen through proteolytic cleavage of the secretory component of pIgR.^285^ Noncanonical NF-κB-inducing kinase (NIK) signaling in DCs is required for pIgR expression in IECs and IgA secretion.^286^ Generally, most secreted IgA (sIgA) is produced in a T cell-dependent manner.^287^ Specifically, bacterial antigen stimulates the migration of IgA^+^ B cells from Peyer’s patches (PPs) to the intestinal stromal layer where IgA^+^ B cells produce IgA and secrete it into the intestinal lumen.^288^ CCL28 indirectly promotes IgA secretion by stimulating the homing of IgA antibody-secreting cells (ASCs).^289^ Gut PCs can also generate IgA through a T cell-independent mechanism. For instance, endoplasmic reticulum stress in IEC elicits the expansion and activation of peritoneal B1b cells in a T cell-independent and microbiota-independent manner, leading to an intensive barrier-protective IgA response.^290^

GF mice exhibit a remarkable reduction in intestinal IgA-secreting cells as well as a failure to generate IgA, which can be rapidly rescued following bacterial colonization, indicating the pivotal stimulatory signals of gut symbiotic bacteria for local sIgA biosynthesis.^287,291^ Indeed, a wide array of studies have demonstrated the sophisticated mechanisms of specific symbionts (such as *B. fragile*,^71^ *SFB.*^274,292^) IL-21 signaling in the small intestine plays an integral role in inducing an IgA-specific response to *SFB*, as deletion of IL-21 receptor diminishes the number of germinal center and IgA^+^ B cells along with a remarkable reduction in small intestine IgA^+^ plasmablasts (PB) and PCs.^293^ *Pediococcus* *acidilactici* *K15*, one of the strains of lactic acid bacteria whose bacterial RNA endows DCs to secrete IL-6 and IL-10, resulting in increased B cell-derived sIgA at mucosal sites in humans.^294^ Intriguingly, crosstalk between gut microbiome and IgA is not limited in the gut. For example, intestinal commensal also exerts evident effects on the serum IgA repertoire. Intestinal colonization of *H. muridarum* in mice induces the development of IgA-secreting PCs in the BM, enhancing serum IgA levels and conferring protection against bacterial sepsis.^295^ Similarly, during the onset of EAE, supplementation of intestinal microbiota that stimulates IgA secretion can facilitate the development of gut-derived IgA^+^ PC and PB. These cells subsequently migrate to the brain and spinal cord from the gut and markedly alleviate the disease through IL-10 secretion.^296^

IgA plays an essential role in preventing host colonization and pathogen invasion that is dependent on multiple mechanisms. IgA fortifies the intestinal physical barrier, where IgA coats and aggregates luminal antigens to avert their direct exposure to the host, preventing potentially detrimental irritation in the mucosal immune system.^287^ sIgA has agglutination potential against invasive pathogens to accelerate pathogen elimination through intestinal peristalsis and mucosa cilia movement or to immobilize the microbiome via downregulating flagella-related gene expression.^297^ Furthermore, IgA-mediated cross-linking causes daughter cells to aggregate, which is conducive to the elimination of pathogens from the gut lumen.^298^ In addition, if infection occurs at the intestinal lamina propria, the formation of IgA immune complexes (IgA-IC) mediates the conversion of immune tolerance into the inflammatory response by selectively augmenting proinflammatory cytokine production by human CD103^+^ DCs.^299^ Notably, microbial-derived ATP impedes the anti-infective effect of IgA in the small intestine.^300^

In addition to counteracting pathogenic infections at mucosal surfaces, IgA can establish and stabilize intestinal commensal colonization and internal symbiosis.^301^ Specifically, the commensal colonization factor system in *B. fragilis* modulates the expression of capsular polysaccharide on the surface of the bacteria to induce close binding to IgA, which makes bacteria themselves enter the intestinal mucus layer and become a stable component of the gut microbiome.^71^

Similarly, heavily glycosylated IgA modulates the expression of mucus-associated functional factor in *Bacteroidetes* by discerning OVA coats of *Bacteroides thetaiotaomicron* (*B. thetaiotaomicron*), which enhances the intestinal colonization of *Bacteroides* and facilitates mutualism with other members of the phylum *Firmicutes*.^302^ IgA deficiency results in dysbiosis, favoring expansion of the gut microbiome with potentially inflammatory properties while inhibiting the growth of anti-inflammatory taxonomic groups.^286,303,304^ Studies of IgA-deficient mice showed more *Firmicutes* with increased *SFB*. Consistent with this, a lack of IgA unexpectedly induces the commensal bacterium *B. thetaiotaomicron*, which typically fails to elicit intestinal inflammation in humans in order to generate increased gene products associated with NO metabolism and to enhance the proinflammatory response in the host.^257^

Interestingly, the IgM antibody isotype partially compensates for IgA deficiency. Furthermore, there is an intriguing probability that varying microbiota engender qualitatively different IgA responses. That is, under the condition of IgA, potentially pathogenic microbes potentially induce compensatory IgM responses, while commensals such as *B. fragilis* potentially display a pathway that particularly elicits IgA rather than compensatory IgM.^303–305^

### The gut microbiome and ILCs

The modulation of the gut microbiome in the innate immune system seemingly extends far beyond classic innate immune cells. ILCs that are deficient of any antigen-specific receptors serve as a newly discovered arm of the innate immune system, and their phenotype and functional plasticity are elicited by different transcription factors.^306,307^ ILC3s, a subset of ILCs, converge in the intestinal tract and lymphoid tissues and establish considerable communication with the gut microbiome and immune cells to form a finely tuned network between the individual and its commensals that support and maintain gut homeostasis.^308^ ILC3s modulate local processes through which mononuclear phagocytes in the lamina propria capture commensal-associated antigens.

ILC3s are well established as pivotal regulators of adaptive immunity. ILC3 deficiency in conventional mice contributes to disruptive adaptive immune responses to the bacterial community.^309^ Specific excision of MHCII expression on RORγt^+^ ILCs leads to intensive proinflammatory Th17 responses against commensals, triggering spontaneous colitis.^309^ As described previously, ILC3-derived IL-22 stimulates Th17 cell differentiation by driving IECs to produce SAA and other factors required for the induction of TH17 cells.^265,268,269^ Instrumental crosstalk has also been depicted between ILC3s and B cells. ILC3-derived lymphotoxin-α (LT-α) and LT-β induce IgA production for microbiota homeostasis in the gut.^310^ The significance of ILC3 cells in host–microbial interactions was clarified in a study in which their depletion and the resulting abrogation of Treg differentiation resulted in the loss of host immune tolerance.^311^ To maintain tolerance to commensal bacteria, MHCII-expressing ILC3 cells elicit the apoptosis of commensal microbiome-reactive CD4^+^ T cells and impede commensal-specific T cell responses.^312^ Microbial sensing and macrophage-generated IL-1β stimulate the secretion of ILC3-derived granulocyte–macrophage colony-stimulating factor, which promotes Treg development and immune tolerance via IL-10 generation by DC and monocytes.^313,314^ ILC3-derived IL-2 also plays a central role in maintaining Treg cells, which in turn orchestrates intestinal homeostasis. In the small intestine, macrophage-generated IL-1β elicits IL-2 production through MyD88- and NOD2-mediated microbial sensing to facilitate Foxp3^+^ Treg cell development; this finding elevated our understanding of the microbiota- and IL-1β-dependent axis by which ILC3 cells control the function and development of Treg cells.^315^ Notably, OX40L is a ligand for OX40 that is expressed in mucosal ILC3 cells and in intestinal Treg cells, thereby serving as a favorable regulator of intestinal Treg homeostasis in Rag1^−/−^ mice.^311^ In addition, Mincle (the Syk-coupled C-type lectin receptor) in intestinal DCs triggers IL-22 generation by ILC3 cells in response to mucosal colonization commensals in the PPs.^316^ Intriguingly, the microbiome–ILC3 partnership also involves glial cells in the lamina propria to accelerate the intestinal steady state. The neurotrophic-factor-expressing glial cells sense bacterial cues to produce neurotrophic factors that stimulate ILC3-derived IL-22 secretion via the neuroregulatory receptor RET, providing the first description of the association between intestinal neurons and innate immune modulation.^317,318^

Consistent with the evidence revealing how ILC3s directly connect to the gut microbiome while interacting intimately with additional immune cells to maintain health, ILC3s are considered to possess anti-inflammatory properties, intestinal epithelial regeneration, and colonization resistance to pathogens, which are largely attributable to microbiota-induced IL-22 generation by ILC3s. ILC3-derived IL-22 limits the availability of iron for pathogenic bacteria and consequently impedes the systemic growth of bacteria.^316^ IL-22 also enhances the expression of the enzyme fucosyltransferase 2 and fucosylation of surface proteins on IECs, which confers protection against enteric pathogens.^319^ IL-22-induced upregulation of epithelial Claudin-2 is conducive to enteric pathogen clearance.^320^ Disruption of IL-23-IL-22 signaling impairs intestinal barrier integrity and allows the growth of pathogenic microbiomes with unique biosynthetic and metabolic capabilities, leading to increased systemic levels of pro-atherogenic metabolites such as LPS and trimethylamine N-oxide to deteriorate atherosclerosis.^321^ LIGHT (the TNF superfamily member)-mediated activation of herpes virus entry mediator in ILC3 induces protective ILC3-derived IFN-γ production to shield against infection by the bacterial pathogen *Yersinia enterocolitica*.^322^ ILC3-derived IL-22 can also cooperate with IFN-γ to confer protection against viral infection.^323^ Additionally, commensal-induced IL-22 production elicits STAT3 phosphorylation in Lgr5^+^ ISCs to enhance epithelial regeneration, leading to proper wound healing.^324,325^ IL-22 is essential for initiation of the DNA damage response following DNA damage and maintains genome stability by activating STAT3/ATM in ISCs, shielding against mutation and tumorigenesis.^326^ By acting through EP4, PGE2 directly induces ILC3 to yield IL-22, conferring a pronounced propensity towards downregulated inflammation.^327^ In a mouse model of *C. rodentium*-induced colitis, CD18 (β2-integrin) deficiency deteriorates the intestine and leads to inflammatory injury, characterized by reduced IL-22 and enhanced systemic bacterial burden. These phenotypes can be reversed via exogenous IL-22 administration, indicating the immunosuppressive effect of IL-22 on intestinal inflammation.^328^ In summary, these results indicate that the significance of ILC3s and derived IL-22 in protecting against pathogen infection, relieving inflammation, and facilitating symbiosis with commensals could depend on crosstalk among the gut microbiome, additional immune cells, IECs, and, surprisingly, with neuro-glial cells to maintain gut homeostasis.

Nevertheless, different conclusions have also been reached regarding whether ILC3-IL-22 signals provide sufficient benefit to the host. In a landmark study, ILC3s aggravated inflammation through the secretion of IL-17 and IFN-γ in mice with *H. hepaticus* infection.^329^ Consistent with this, gut macrophages with IL-10 receptor deficiency stimulate IL-22 production by ILC3s in a microbiome-dependent manner. ILC3-derived IL-22 further triggers intestinal inflammation through detrimental neutrophil recruitment.^330^ The significantly reduced levels of IL-22 are associated with enhanced colonization resistance against *S. typhimurium* in mice.^331^ Similarly, in both a mouse model with intestinal fibrosis and patients with CD, the IL-23/IL-22 axis in the gut promotes intestinal fibrosis in a T cell- or B cell-independent manner. Cell experiments further confirmed that IL-22 cooperates with TGF-β to accelerate fibrosis, indicating the therapeutic potential of targeting IL-22 in intestinal fibrosis.^332^ The IBD-associated adherent gut microbiome stimulates intestinal CX3CR1^+^ MNP to produce TL1A that mediates OX40L expression in MHCII^+^ ILC3s, giving rise to the amplification of antigen-specific T cells and pathogenic Th1 and thus driving chronic colitis.^333^ In favor of the pathogenic role of ILC3s in gastrointestinal inflammation, IL-22 can synergize with *H. pylori* to upregulate matrix metallopeptidase-10 in the gastric mucosa via the extracellular-signal-regulated kinase pathway, worsening gastric inflammation and supporting *H. pylori* colonization.^334^ In particular, Tregs are endowed with the capacity to confer protection against ILC3-induced colitis.^335^ Latent activation gene 3-expressing Treg cells diminish IL-1β and IL-23 generation by CX3CR1^+^ macrophages, contributing to disruptive IL-22 generation by ILC3s and thus mitigating disease.^335^

ILC3s, considered the best-characterized ILC lineage, have become a focus of the immune field because of their diversified functions in modulating the delicate balance between immune tolerance to nonpathogenic antigens and swiftly and vigorously withstanding potential pathogenic stimuli, which is largely attributed to their interaction with intestinal immune cells and the gut microbiome. Additional studies are warranted to provide a more in-depth characterization of the biological behavior of ILC3s and the influence of relevant cytokines (including IL-23, IL-1β, IL-22) on ILC3.^336^

### The effects of the gut microbiome on tumorigenesis and the progression of extraintestinal cancers

In 2018, over 18.1 million individuals were newly diagnosed with cancer, affecting 9.6 million lives globally; thus, cancer can be considered the single most significant obstacle to extending life expectancy.^337^ The global burden of cancer is anticipated to rise as increasing populations are exposed to risk factors. Epidemiologic studies support that genetic susceptibility,^338^ environmental factors,^38,339–341^ metabolic disorders,^342^ and/or chronic infection^343^ contribute to the onset of cancer. As our understanding of the gut microbiome grows, we have recently come to appreciate intriguing evidence suggesting that the gut microbiome plays a momentous role in tumorigenesis and progression, especially extraintestinal neoplasms, such as hepatocellular carcinoma,^32^ mammary cancer,^33^ pancreatic cancer,^344^ and melanoma.^35^ We seek to summarize the latest data demonstrating alterations in the intestinal microbiota of patients with the above-mentioned extraintestinal cancers (Table 2).

**Table 2:** Studies exploring the gut microbiome alterations in patients with extraintestinal cancers

Tumor type	Cases with cancer, controls, *n*	Sample type and method for detection	Alterations of gut microbiome	Findings	Ref.
Pancreatic tumor	32 patients with PDA vs. 31 healthy controls	Fecal specimens, 16S rRNA gene sequencing	*Proteobacteria* and *Synergistetes* ↓; *Euryarchaeota* ↑	Increased feces microbiome in patients	[349]
Liver cancer	15 HCC patients vs. 15 non-HCC patients	Stool specimens, *E. coli* counts	*E. coli* ↑	Potentially drive tumorigenesis	[370]
	75 HCC patients vs. 75 healthy controls	Fecal samples,16S rRNA gene sequencing	Butyrate-producing genera (including *Faecalibacterium*, *Clostridium* IV) ↓; LPS-producing genera (such as *Klebsiella* and *Haemophilus*) ↑	Potential biomarkers for early diagnosis of HCC	[362]
	21 NAFLD-related HCC vs. 20 healthy controls	Stool specimens,16S rRNA gene sequencing	*Bacteroides* and *Ruminococcaceae* ↑; *Bifidobacteria* ↓	Correlate with systemic inflammation and tumorigenesis	[371]
	68 HCC patients vs. 18 healthy controls	Stool specimens,16S rRNA gene sequencing	Proinflammatory bacteria *Proteobacteria*, *Enterobacter*, *Haemophilus* ↑	Degree of dysbiosis reflects the severity of HCC	[372]
	25 cirrhotic patients with HCC vs. 25 cirrhotic patients without HCC	Fecal stool samples,16S rRNA gene sequencing	*Fusobacterium*, *Leuconostocaceae* ↓; *Butyricimonas*, *Erysipelotrichaceae*, ratio *Bacteriodes*/*Prevotella* ↑	Linked to inflammation and potential HCC biomarker	[373]
	35 individuals with HBV related HCC vs. 22 individuals with non-HBV non-HCV-related HCC	Stool samples, 16S rRNA gene sequencing	NBNC-HCC: proinflammatory bacteria (*Escherichia–Shigella*, *Enterococcus*) ↑; SCFA-producing bacteria (*Faecalibacterium*, *Ruminococcus*, *Ruminoclostridium*) ↓	Different gut microbiome composition corresponds to different pathological types	[374]
Breast cancer	48 postmenopausal BC patients vs. 48 controls	Stool samples, 16S rRNA gene sequencing	*Clostridiaceae*, *Faecalibacterium*↑; *Dorea* and *Lachnospiraceae* ↓	Altered composition and low diversity of gut microbiome in patients	[403]
	44 postmenopausal BC patients vs. 46 postmenopausal healthy controls	Feces samples, 16S rRNA gene sequencing	*Escherichia coli*, *Klebsiella*, *Prevotella amnii*, *Enterococcus gallinarum*, *Actinomyces*, *Shewanella*, *Putrefaciens* ↑; *Eubacterium eligens* and *Lactobacillus vaginalis, Acinetobacter radioresistens* and *Enterococcus gallinarum* ↓	The composition and functions of the gut microbial community differ between postmenopausal BC patients and healthy controls	[402]
	48 postmenopausal BC patients vs. 48 postmenopausal healthy controls	Fecal samples, 16S rRNA gene amplicon sequencing	Reduced α-diversity; altered composition of both their IgA-positive and IgA-negative fecal microbiome	BC cases have estrogen-independent associations with the IgA-positive and IgA-negative gut microbiota	[401]
	31 patients with early-stage BC	Fecal samples, 16S rRNA gene amplicon sequencing	*Firmicutes*, *Faecalibacterium prausnitzii*, and *Blautia* differ according to the patient’s BMI and clinical stages	Gut microbiome composition in BC patients differs according to clinical characteristics and BMI	[404]
Pulmonary cancer	41 patients with lung cancer vs. 41 healthy volunteers	Fecal samples, 16S rRNA gene amplicon sequencing	*Bacteroides*, *Veillonella*, *Fusobacterium* ↑; *Escherichia*, *Shigella*, *Fecalibacterium*, *Enterobacter*, and *Dialister* ↓	An altered bacterial community in patients with lung cancer	[439]
Prostate cancer	64 patients with prostate cancer vs. 41 individuals without cancer	Rectal swab samples,16S rRNA amplicon sequencing	*Bacteroides* and *Streptococcus* ↑	An altered bacterial community in patients with prostate cancer	[435]
	30 patients with or without oral androgen receptor axis- ies (ATT)	Rectal swab samples,16S rDNA amplicon sequencing	Patients receving ATT: *Akkermansia muciniphila*, *Ruminococcaceae* spp. ↑	There are measurable differences in the bacterial community of men receiving oral ATT	[436]

### Gut dysbiosis and pancreatic cancer

Pancreatic cancer has emerged as the seventh leading cause of cancer-related deaths worldwide. The incidence of pancreatic cancer has risen annually, and ~458,918 new cases have been diagnosed worldwide in 2018. This type of cancer is interrelated with the gut microbiome and has an extremely unfavorable prognosis, with a 5-year survival rate of merely 9%.^337,345^ The onset and development of pancreatic cancer is partially influenced by certain risk factors, such as obesity, diabetes, metabolic syndrome, and chronic pancreatitis, all of which have been identified to be associated with gut dysbiosis.^102,126,345,346^ Consequently, gut dysbiosis has been postulated to facilitate the development of pancreatic cancer by extraintestinal mechanisms.

The upper gastrointestinal microbiome may be implicated in pancreatic cancer due to adjacent anatomical locations.^347^ A meta-analytical study has highlighted that *H. pylori* infection, a common pathogen that colonizes in the upper digestive tract, corresponds to an increased risk of pancreatic cancer;^348^ however, a nested case–control study using 448 pancreatic cancer cases revealed no association between the two,^349^ and several studies even drew the opposite conclusion.^350^ The elimination of confounding factors is one strategy that could untangle these inconsistent and contradictory associations.

The microbiome is truly subsistent in the human pancreatic tumor microenvironment.^344,351–353^ Nevertheless, the intrapancreatic microbial composition potentially fails to differentiate pancreatic tumors from nontumor tissues.^344^ Compared with pancreatic adenocarcinoma (PDAC) patients with short-term survival (STS), PDAC patients with long-term survival (LTS) are endowed with higher α-diversity in the tumor microbiome.^353^ Furthermore, the intratumoral microbiome (*Pseudoxanthomonas*, *Streptomyces*, *Saccharopolyspora*, *Bacillus clausii*) is demonstrated as a predictive signature of LTS and is also associated with a relative abundance of intratumoral CD8^+^ T cells.^353^ Similarly, intratumor bacteria (mainly *Gammaproteobacteria*) were found to be present in 76% of 113 patients with pancreatic cancer. *Gammaproteobacteria* are considered a representative bacterial genera in the gut and are abundant in the duodenum, indicating that retrograde bacterial migration from the duodenum to the pancreas may be a source of tumor-associated bacteria.^351^ The migration of the gut microbiome to the pancreatic tumor microenvironment has been revealed through fluorescent-labeled microbes and 16S sequencing.^352^ In addition, the richness of the *Fusobacterium* genus is an independent prognostic factor for adverse clinical outcomes.^354^ Future investigations are necessary to explore whether the existence of such an intratumor microbiome is causative or merely an outcome of the dysfunctional tumor environment, and more evidence is needed to confirm that these identified microbiome species are not simply indicative of contamination.^34^ The gut microbiome can migrate into the pancreas, and the relative abundance of the microbiome is remarkably higher in pancreatic tumor specimens than in normal pancreas specimens in both mice and humans.^352^ Interestingly, the altered composition of the gut microbiome related to dysbiosis is not consistent with alterations in the intratumoral microbiome composition. Specifically, the commensal beneficial bacteria *B. pseudolongum* in the gut are continuously enriched along with cancer progression. Furthermore, repopulating GF mice with fecal bacteria derived from tumor-bearing hosts or with *B. pseudolongum* exerts concerning effects, namely, the induction of tumor progression and intratumoral immunosuppression.^352^ Patients with pancreatic cancer and chronic pancreatitis are characterized by increased serum levels of antibodies against the capsular polysaccharide of *Enterococcus faecalis* (*E. faecalis*) compared with healthy volunteers, suggesting that infection with *E. faecalis* is involved in the development of pancreatitis-associated cancer.^355^ Similarly, another study that conducted a microbiological investigation of bile from female patients with pancreas head carcinoma (PHC) showed a remarkably positive correlation between the abundance of *Bactibilia* and PHC. Conversely, *E. coli* and *Pseudomonas* spp. are strongly and negatively associated with PHC.^356^ Notably, patients with intraductal papillary mucinous neoplasms with high-grade dysplasia are characterized by high levels of IL-1β and intracystic bacteria that exhibit cooccurrence with abundant oral bacterial taxa, including *F. nucleatum* and *Granulicatella adiacens*, indicating the role of oral bacteria in cystic precursors to pancreatic cancer.^357^ Indeed, as has been reported previously, unsatisfactory oral health conditions, pathogenic oral microbiomes (such as *Porphyromonas gingivalis* and *Aggregatibacter actinomycetemcomitans*), periodontitis, and tooth dislocation are correlated with a higher risk of pancreatic cancer.^350,358^

Antibiotics-mediated depletion of certain detrimental microbiomes attenuates neoplastic progression.^344,352^ In the mouse xenograft model, oral administration of antibiotics elicits notably decreased progression and growth of sterile neoplastic grafts, indicating that the gut microbiome affects pancreatic cancer in a long-distance manner independent of intrapancreatic/intratumoral microbiota or the matrix microenvironment.^344^ Additional mechanisms are potentially attributed to altered immune responses within the tumor microenvironment owing to gut microbiome alterations. Initially, gut microbial and metabolite-induced inflammation perpetually enables the development of pancreatic cancer, as has been reported in animal models, and LPS generated from Gram-negative bacteria can act through TLR4 to enable the activation of proinflammatory NF-κB and mitogen-activated protein kinase signaling, thus facilitating pancreatic carcinogenesis.^359^ Furthermore, the accumulation of certain intestinal and intrapancreatic bacteria fosters a tolerogenic immunosuppressive microenvironment conducive to cancer development and resistance to immunotherapy. As an instance, antibiotic-mediated bacterial ablation induces a diminished number of immunosuppressive CD206^+^ M2-like tumor-associated macrophages (TAMs) while increasing tumor-protective M1-like TAMs, reinforcing Th1 differentiation of CD4^+^ T cells and CD8^+^ T cell activation, thereby enhancing anti-tumor immunity.^352,360^ Similarly, antibiotic-mediated elimination of the gut microbiome significantly enhances the levels of IFN-γ-producing Th1 cells concomitant with diminished IL-17A- and IL-10-producing T cells, thereby weakening the pancreatic tumor burden.^361^ The clinical response to chemotherapy and immunotherapy is also modulated by the microbiome. Specifically, the intratumor bacteria *Gammaproteobacteria* is endowed with the capacity to metabolize the chemotherapeutic drug gemcitabine into its inactive form, thereby contributing to drug resistance, which could be reversed by the antibiotic ciprofloxacin treatment.^351,352^ In the same vein, gut microbiome depletion by oral antibiotics is responsible for upregulating programmed cell death protein 1 (PD-1) expression to intensify checkpoint-targeted immunotherapy.^352^

Tumor stroma-producing pancreatic stellate cells (PSCs) have been well established to play a central role in the pancreatic tumor microenvironment.^350^ The assumption that the gut microbiome facilitates tumor progression by activating PSC merits further experiments and exploration. Further studies are warranted to address whether successful modulation of the gut microbiome potentially enhances the therapeutic effect of pancreatic cancer. Additional human studies are required prior to clinical application.

### Gut dysbiosis and hepatocellular carcinoma

Liver cancer is the second leading cause of cancer-related deaths globally, and HCC accounts for ~90% of all primary liver cancer cases.^362^ Liver cancer incidence is rising faster than any other cancer in both men and women. It is estimated that in the United States, there will be ~42,030 new liver cancer cases and 31,780 deaths in 2019.^363^ HCC is usually diagnosed at an advanced stage, with its poor prognosis (overall ratio of mortality to morbidity is 0.95) in part due to the absence of early specific symptoms as well as the lack of effective and accurate diagnostic biomarkers.^364^ The overall median survival of untreated patients with HCC is 4 months, ranging from 2 months for advanced cancer to 14 months for early-stage cancer.^365^

Hepatocarcinogenesis is associated with one or more risk factors, principally driven by a vicious cycle of long-term infection with hepatitis B virus or hepatitis C virus, accounting for 60–85% of HCC cases.^363,366,367^ In recent years, increasing evidence suggests that metabolic disorders (including obesity, type 2 diabetes or hypertension/dyslipidemia, nonalcoholic fatty liver disease, and metabolic syndrome) will be becoming the most important risk factors for liver cancer, potentially overtaking other etiologies in some areas of the world.^368,369^ Approximately 75% of the nutrient-rich blood flowing through liver comes from the gut through the portal vein; thus, the liver is consistently exposed to components and metabolites of the gut microbiome.^186^ Consequently, awareness is growing that gut dysbiosis plays a pivotal role in hepatocarcinogenesis and the progression of HCC.^370^ Moreover, in a prospective study, the utilization of antibiotics targeting anaerobes exerted detrimental effects on both the progression-free survival (PFS) and overall survival (OS) of patients with HCC who underwent chemotherapy. In contrast, the fecal levels of anaerobes *Blautia* are positively associated with the prognosis of such patients, indicating the influence of antibiotic-mediated gut dysbiosis on HCC treatment and the need for caution concerning the overuse of broad-spectrum antibiotics with anti-anaerobe activity in patients with HCC.^371^

**Fig. 7:**
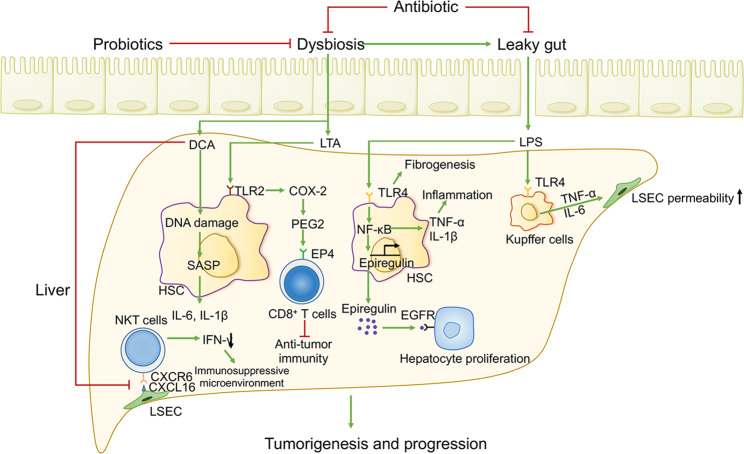
The underlying mechanisms by which the gut microbiome and its metabolites influence hepatocarcinogenesis and progression. Dysbiosis and a leaky gut facilitate hepatocarcinogenesis and progression through distinct mechanisms. Microbial-derived LPS can worsen liver inflammation and fibrosis and favor hepatocyte proliferation in a TLR4-dependent manner. DCA induces DNA damage and the SASP in HSCs and synergizes with LTA to weaken the anti-tumor activity of CD8^+^ T cells. Moreover, DCA downregulates the accumulation of CXCR6^+^ NKT cells in the hepatic tumor microenvironment, which is conducive to hepatic tumor growth

Currently, accumulating clinical studies have uncovered that there are significant differences in intestinal microbial composition and function between HCC patients and healthy counterparts, and a consensus has been reached: HCC patients are generally characterized by an enhanced abundance of proinflammatory bacteria along with relatively diminished levels of SCFA-producing bacteria.^364,372–376^ These reports have set the stage for the potential development of noninvasive methods for the early diagnosis of HCC through gut microbiome-associated biomarkers. Based on the clinical evidence that aberrant alterations in the functions and composition of the gut microbiome occur in patients with HCC, scientists have generated much enthusiasm for the exact mechanisms by which the gut microbiome exerts impacts on the onset and progression of HCC (Fig. 7). For example, *H. hepaticus*, a pathogenic gut bacterium that frequently colonizes the cecal and colonic mucosa of mice, has been detected in human HCC tissue specimens.^377–380^ Experimental studies have shown that cytolethal distending toxin, one of the functional subunits of *H. hepaticus*, potentiates liver tumor development by activating the Wnt/β-catenin and NF-κB pathway, promoting senescence and endoreplication and increasing p21 and Ki-67 expression.^381–383^ In addition, the overgrowth of *E. coli* in the gut may trigger hepatocarcinogenesis.^372^

The liver can exhibit a range of pathological changes, such as hepatic inflammation, fibroblast proliferation, and tumorigenesis following the sustained stimulation of microbial-derived metabolites or components such as LPS.^384^ Both mice and patients with HCC characteristically display failing intestinal barrier integrity and gut dysbiosis, as well as high levels of circulating LPS, which serves as an inducer of inflammation to expedite liver fibrosis.^384,385^ In mice with dimethylnitrosamine-induced hepatocarcinogenesis, due to the failing intestinal barrier, LPS can enter the liver and bind to TLR4 on hepatocytes, hepatic stellate cells (HSCs) and Kupffer cells, which facilitates tumor progression through multiple mechanisms.^386,387^ In HSCs, the LPS/TLR4 pathway triggers upregulation of the hepatomitogen epiregulin, which belongs to the epidermal growth factor family and has a potent mitogenic effect on hepatocytes, thereby inhibiting hepatocyte apoptosis.^386,388^ Additionally, LPS/TLR4 activation in Kupffer cells elicits the secretion of proinflammatory mediators (such as TNF-α and IL-6) and further increases the permeability of hepatic sinus and compensatory hepatocyte proliferation, thereby contributing to HCC exacerbation.^387^ Furthermore, the overexpression of IL-6 activates JAK/STAT3 and facilitates the polarization of IL-10-producing M2 macrophages, potentially triggering invasion, metastasis, and drug resistance in HCC.^389,390^

Interestingly, in mice with bile duct ligation, dehydroandrographolide can suppress LPS/TLR4 activation in Kupffer cells and HSCs to inhibit LPS-stimulated inflammatory responses.^391^ Similarly, the commensal microbial-derived granisetron, a 5-HT3 receptor antagonist, exerts anti-inflammatory effects on the liver by inhibiting the overexpression of proinflammatory cytokines in macrophages, which is dependent on the suppressive effect on LPS/TLR4-mediated NF-κB activation and phosphorylated-p38 (P-p38) accumulation in macrophages.^391^

As mentioned previously, obesity has been identified as a major risk factor for HCC. In mice with nonalcoholic steatohepatitis-induced HCC driven by the combination of DMBA and HFD (DMBA–HFD), there is a remarkable increase in Gram-positive bacteria (G^+^ bacteria), especially *Clostridium* clusters, as well as serum levels of DCA that induce DNA damage by mediating ROS production.^184,185^ The enterohepatic circulation of DCA elicits DNA damage and a senescence-associated secretory phenotype (SASP) in HSCs as well as the resulting secretion of inflammatory and cancer-promoting factors (such as IL-6 and IL-1β), thus leading to obesity-related HCC development.^184^ In addition, lipoteichoic acid, the prime cell wall components in G^+^ bacteria, synergize with DCA to perpetuate the SASP in HSCs, to activate NF-κB via binding to TLR2 and to upregulate the expression of cyclooxygenase 2 (COX2). COX2-mediated PGE2 suppresses anti-tumor immunity by binding to the PTGER4 receptor expressed on CD8^+^ cells.^392^ These results suggest that the DCA–SASP axis in HSCs exerts a significant effect on obesity-related HCC development. DCA also activates the mTOR pathway in hepatocytes and induces the production of TNF-α and IL-1β, as well as the infiltration of inflammatory macrophages, which in turn promotes the development of HCC.^393^ Surprisingly, SCFAs in cooperation with abnormal secondary bile acid profiles can enhance HCC susceptibility in dysbiotic mice.^32^ In addition, the enterohepatic circulation of secondary bile acids also modulates hepatic NKT cell populations to influence HCC. More precisely, *Clostridia*-derived secondary bile acids such as LCA suppress the recruitment of IFN-γ-producing CXCR6^+^ NKTs to the hepatic tumor microenvironment by suppressing the expression of the chemoattractant C-X-C motif chemokine 16 (CXCL16) by liver sinusoidal endothelial cells, which fosters an immunosuppressive tumor-associated microenvironment and thus promotes tumorigenesis in animal models.^186^ This finding suggests that regulating bile acid composition by individualized manipulation of the gut microbiome may be a novel and attractive therapeutic strategy option for patients with liver cancer. However, whether these animal findings can be applied to humans remains unclear. The immune system and the composition of gut microbiota differ between mice and humans. NKT cells constitute up to 30% of liver lymphocytes in mice; in contrast, only ~1% of hepatic lymphocytes are NKT cells in humans. In addition, the composition of bile acids varies between mice and humans. Moreover, reinforcing the effector function of intrahepatic lymphocytes in patients potentially triggers side effects such as inflammation and autoimmune reactions.^394^ Consequently, considering the differences between humans and mice, comprehensive analysis of immunity and metabolism in human liver tissue is indispensable for future clinical applications. In addition to primary HCC, in mice with CRC, an unhealthy gut microbiome stimulates the secretion of metastasis-related secretory protein cathepsin K, which potentiates the development of CRC-derived metastatic hepatic cancer via M2 polarization of TAMs.^395^ Intriguingly, in the setting of gut dysbiosis, tuft cell-derived IL-25 enters the liver and then triggers macrophage activation, facilitating hepatocarcinogenesis and the migration of HCC cells by chemokine CXCL10.^396^

Gut dysbiosis is a pivotal stepexpress interleukin-10 receptor and in the tumorigenesis and progression of HCC. This dysbiosis is facilitated by various pathways, including the production of tumor-accelerating and SASP-accelerating metabolites such as DCA derived from the dysbiotic microbiome, the increase in hepatic contact with microbiome-associated molecular patterns (MAMPs) such as LPS and the reduction in the accumulation of CD8^+^ T cells and NKT cells in the hepatic microenvironment, as well as consistent hepatic exposure to tumor-promoting cytokines such as IL-25. The utilization of inhibitors to target such microbial-derived metabolites or gut-derived detrimental cytokines represents a potential therapeutic intervention.

### Gut dysbiosis and breast cancer

Breast cancer occurs in mammary gland epithelial tissue, and it is the most common malignant neoplasm among women. There are ~3.86 million newly diagnosed female breast cancer cases in America in 2019.^397^ Because of the extensive use of mammography for breast cancer screening as well as the improvement of personalized medication,^398^ the overall 5-year relative survival rate of female breast cancer patients in America has reached 90.3%, but international differences vary widely and are as low as 66.1% in developing countries.^399^ Hereditary factors such as a personal or family history of breast or ovarian cancer and genetic mutations (in BRCA1, BRCA2, and other novel susceptibility genes for breast cancer, such as SNX32, ALK and BTN3A2) are responsible for 5 to 10% of breast cancer cases.^337,400^ Its rising incidence is attributed to nonhereditary risk factors that are closely associated with menstruation of an inappropriate duration, reproduction, and supplementation with exogenous hormones, as well as stress.^337,401^ Notably, there is a strong and significant positive correlation between obesity and the incidence of breast cancer; the risk of breast cancer increases by 12% with every 5-unit increase in body mass index.^402^

There are a plethora of data connecting the gut microbiome to obesity in the host, and some clinical studies have also demonstrated the association between gut microbiome composition and breast cancer condition. Patients with breast cancer are characterized by diminished gut microbiome diversity and increased levels of *Clostridiales* compared with controls. Variation in the fecal microbiome with clinical stages and histoprognostic grades is also demonstrated.^403–406^ Antibiotic-mediated intervention is associated with a lower gut microbiome diversity and increased breast cancer risk, as well as the risk of recurrence.^407–412^ Oral administration of cephalosporin further exacerbates the tumor-induced reduction in gut microbiome diversity, accompanied by decreased levels of butyrate-producing bacterial groups, and accelerates breast tumor progression in an animal model.^412^ Similarly, antibiotic-mediated preexisting commensal dysbiosis remarkably increases tumor metastasis and worsens mammary tissue inflammation in mice with hormone receptor-positive (HR^+^) breast cancer, which is largely attributable to dysbiosis-induced accumulation of the structural protein collagen and macrophages within the breast tissue.^413^ These animal studies provide correlative support for the notion that antibiotic utilization is causally responsible for breast cancer development. Nevertheless, antibiotics are merely a conventional strategy to induce chronically unhealthy microbiomes in mice. Antibiotics are not dangerous and should not be absolutely avoided by cancer patients or anyone who requires them. Mice, after all, are not humans, and further studies are warranted to address whether a causal association exists between long-term antibiotic utilization and cancer patient outcomes in clinical practice.^413^

Studies in animal models of breast cancer have also demonstrated that alterations in the gut microbiome exert long-distance effects on the onset and development of breast cancer via mechanisms that are potentially involved in systemic immune modulation, including immune cell infiltration, fibrosis, and tumor-associated inflammation.^414–416^ For example, infection with *H. hepaticus* in mice facilitates the generation of neoplastic lesions in mammary glands.^414^ Tumorigenesis is potentially initiated through activation of systemic inflammation and the trafficking of systemic immune cells to mammary tissues.^415^ Another explanation is that pathogen-induced compromised intestinal epithelial barrier and gut leakiness contribute to translocation of *H. hepaticus* to mammary tissue along with localized proinflammatory response, thus increasing the risk for cancer,^415^ indicating the profound effects of gut homeostasis on host health. In the setting of *H. hepaticus*-induced cancer, enhanced levels of neutrophils are considered a dominant factor in breast cancer initiation and development.^414^ Clinically, the OS of breast cancer is negatively associated with the neutrophil-to-lymphocyte ratio (NLR),^417^ and there is also a positive correlation between NLR and the risk of late relapse.^418^ Furthermore, TLR5 recognition of the gut microbiome also exacerbates systemic tumor-promoting inflammation and supports the immunosuppressive tumor microenvironment through IL-6 upregulation, mobilization of myeloid-derived suppressor cells, and induction of suppressive galectin-1-producing γδT cells.^416^ In contrast, during homeostasis, gut microbiome-triggered CD4^+^CD25^+^ Treg cells can maintain immune homeostasis and significantly inhibit breast cancer.^419^ Supplementation with beneficial bacteria *L. reuteri* in mice protects the host against mammary carcinogenesis by inducing Treg cells and downregulating the levels of proinflammatory cytokines and cells.^420^ However, under proinflammatory conditions, an attenuated Treg-mediated inhibitory loop renders carcinogenic consequences of enhancive IL-6 and IL-17, resulting in more frequent inflammation-associated distal cancers.^419^ Overall, these studies demonstrate that the gut microbiome exerts an array of different effects on systemic innate and adaptive immunity to affect breast tumor formation and progression.

Gut microbial metabolites enter the systemic circulation and are transferred to target cells, thus modulating the biological behavior of breast cancer. One of the best-characterized examples is microbiome-derived cadaverine, which ameliorates breast cancer by suppressing the proliferation, metastases, and aggressiveness of tumor cells, as well as epithelial-to-mesenchymal transition (EMT).^421^ Cadaverine also initiates the metabolism of tumor cells that have shifted towards glycolysis by decreasing cellular oxygen consumption.^421^ Additionally, microbial-derived LCA is significantly diminished^422^ and is negatively correlated with the Ki-67 index in breast cancer.^423^ The concentrations of LCA (<1 μM) in breast tissue suppresses cancer cell growth, VEGF generation, and metastatic potential partly in a TGR5-dependent manner.^422^ LCA also restrains lipogenesis^33^ and triggers pro-apoptotic effects on breast tumor cells under the condition of supraphysiological concentrations (over 1 µM).^33,424,425^ Furthermore, SCFAs, particularly butyrate, have received considerable attention because of their HDAC-inhibitory role in mediating cancer cell death.^73,426^ SCFAs are sufficient to decrease tumor burden via multiple distinct mechanisms, including cell cycle arrest and induction of apoptosis through ROS generation and mitochondrial impairment,^426^ weakening invasive phenotypes in breast tumors in a FFAR2- and FFAR3-dependent manner.^427^

It has been widely acknowledged that estrogens are pathogenic in HR^+^ breast cancer. A group of human gut organisms, namely, an aggregate of intestinal bacterial genes whose products can metabolize estrogens, has been defined as the estrobolome.^428^ The conjugated estrogens secreted in bile are first deconjugated by β-glucuronidase of the gut microbiome (constituents of the “estrobolome”) and are subsequently reabsorbed into the peripheral circulation. Circulating estrogens interact with target tissues such as breast tissue to facilitate cellular growth and proliferation, thus contributing to the initiation and promotion of neoplastic growth.^429^ Phytoestrogens (also known as dietary estrogens) are plant-derived compounds that act on estrogen receptors to exert estrogenic or/and antiestrogenic effects due to their structural similarity with endogenous estrogens.^430^ Nevertheless, in contrast to estrogen, phytoestrogens are endowed with protective properties against breast cancer by inhibiting estrogen synthesis and metabolism, as well as antiangiogenic and antimetastatic effects.^431,432^ Enterolactone (EL), a type of phytoestrogen, is the intestinal microbial fermentation product of dietary lignans. Epidemiological studies have revealed that higher dietary intake of EL precursors and higher serum concentrations of EL are correlated with decreased breast cancer risk and mortality, as well as more satisfactory survival time, particularly in postmenopausal breast cancer patients.^433^ In animal experiments, EL exerts its anti-breast cancer functions by inhibiting NF-κB-mediated inflammation,^434^ TGF-β-induced EMT,^435^ and cancer cell viability.^436^

Collectively, the gut microbiome potentially affects carcinogenesis through its far-reaching impacts on estrogen metabolism, energy metabolism, and the anti-tumor function of the immune system.

### Additional extraintestinal cancers and the gut microbiome

The alterations in the gut microbiome also potentially contribute to distal effects on other extraintestinal tumors, such as lung carcinoma, prostate cancer, and melanoma, through modulation of systemic metabolism/immune. For example, a consortium of 11 bacterial strains, including *B. rodentium*, establishes anti-tumor immunity and confines melanoma growth when inoculated into GF mice.^35^ There are discrepancies in the intestinal microbial composition and metabolic functions between patients with and without prostate cancer;^437–440^ for instance, the aberrant metabolome of individuals with prostate cancer is characterized by the presence of high levels of bacteria associated with carbohydrate metabolism pathways while lacking bacteria associated with folate production.^437^ Compared with male patients with prostate cancer, men without prostate cancer are characterized by higher α-diversity in the gut microbiome.^438^ There are measurable differences in the gut microbiome composition between prostate cancer patients undergoing oral androgen receptor axis-targeted therapies and those who are not.^438^ The knowledge and understanding of the effects of the gut microbiome on the occurrence and development of lung carcinoma is extremely limited. A clinical trial has shown an immediate association between gut microbiome and lung cancer. That is, *Bacteroides*, *Veillonella*, and *Fusobacterium* are increased in fecal samples in lung cancer patients compared to healthy individuals.^441^ Gut dysbiosis potentially triggers lung cancer proliferation through diminished levels of butyrate.^441^ Bacterial alterations have also been revealed in lung cancer tissues, although their significance remains indistinct. *Streptococcus* and *Prevotella* are enriched in pulmonary tumor tissue,^442^ and the genera *Veillonella* and *Megasphaera* can potentially serve as biomarkers for lung cancer with satisfactory sensitivity and specificity.^443^ However, whether it is a causative agent or a bystander to some additional process remains unclear. Mechanically, the local pulmonary microbiome activates IL-17-producing Vγ6^+^Vδ1^+^ γδ T cells through Myd88-dependent IL-1β and IL-23 secretion, thus promoting inflammation-associated lung adenocarcinoma.^444^ With improvements in tumorigenesis models and more advanced detection techniques for bacteria and their proteins, as well as microbial-derived metabolites, the significance of such microbiomes will become clear. As described below, changes to the gut microbiome influence the response to immune checkpoint inhibitor (ICI) therapy in renal cell carcinoma (RCC), melanoma, and lung carcinoma, as well as HCC.

### Microbiome-mediated effects on cancer immunotherapy

The immune response of T cells is modulated partly by negative regulatory pathways (also known as immune checkpoints). Two such checkpoints, PD-1 and cytotoxic T-lymphocyte protein 4 (CTLA-4, also known as CD152), have attracted widespread attention. Under pathological conditions, CTLA-4 and PD-1, which are expressed on activated T lymphocytes, bind to the corresponding ligand B7 molecule on APCs and PD-L1 expressed on the tumor cells, contributing to an inhibitory immune signal that refines the activity of T cells and assists tumors in evading immunosurveillance.^445,446^

Notably, the precise mechanisms underlying PD-1/PD-L1-mediated tumor immunosuppression remain under investigation. In addition to being expressed on tumor cells, PD-L1 is expressed on the exosomes released by tumor cells. PD-L1-expressing exosomes secreted by tumor cells can enter the peripheral circulation and further suppress the function of circulating CD8^+^ T cells in both patients with metastatic malignant melanoma and tumor-bearing mice models.^447^ Thus, anti-CTLA-4 monoclonal antibody (anti-CTLA-4 mAb) and anti-PD-1/PD-L1 mAb (anti-PD-1/PD-L1 mAb) that target immune checkpoint molecules have been considered remarkably promising anticancer drugs. Anti-PD-1/PD-L1 mAb specifically disrupts the interaction between PD-1 and PD-L1 and thus induces T cell-mediated tumor cell death. PD-1 immune checkpoint blockers can activate tumor-infiltrating CD8^+^ T cells, which stimulate tumor-infiltrating DCs to generate IL-12 by releasing IFN-γ. DC-derived IL-12 enhances the capacity of Teff cells to eliminate malignant cells.^448^ Anti-CTLA-4 mAb is potentially endowed with different properties. For instance, mouse experiments have shown that the suppressive effect of anti-CTLA-4 mAb on tumor progression is ascribed to selective depletion of intratumoral Foxp3^+^ Treg cells rather than to conventional mechanism by which this mAb functionally blocks the interaction between CTLA-4 and B7.^449–451^ However, some controversy remains over the mechanisms of anti-CTLA-4 mAbs in the inhibition of tumor progression because two anti-CTLA-4 mAbs (both ipilimumab and tremelimumab) have been demonstrated to augment the infiltration of intratumoral CD4^+^ T and CD8^+^ T cells without significant alteration or depletion of Fopx3^+^ Treg cells in the tumor microenvironment in patients with melanoma.^452^ It remains to be determined what additional pathways potentially participate in the inhibitory effect of the above monoclonal antibodies on tumor progression and what other molecular types are involved in the immunosuppressive effect on the tumor microenvironment.

It has been well proven that five anti-PD-1/PD-L1 mAbs and anti-CTLA-4 mAbs can successfully treat various cancers owing to improvements in OS in comparison to traditional chemotherapies. Notably, although the laudable achievement of ICIs has formed a novel paradigm in tumor immunotherapy, only a minority of patients respond to immunotherapy and achieve dramatic remissions. However, a host of patients exhibits heterogeneous and unendurable responses to these therapies. The effective response rates to anti-CTLA-4 mAb and anti-PD-1 mAb are ~20 and 25%, respectively, in patients with melanoma.^453,454^ Similarly, additional statistics show that as a single agent, ICIs have response rates in the range 10–35%, which is associated with a limited number of tumor-infiltrating Teff cells, checkpoint disruption, and persistent resistance to checkpoint inhibition.^455^ Therefore, there is an urgent need to combat therapeutic resistance and identify biomarkers that can be used to predict the response to ICIs. The high heterogeneity to ICI therapy in cancer patients can be partially explained by differences in gut microbiome composition, with compelling evidence suggesting that gut microbiome and even specific key bacterial taxa potentially contribute to interindividual variation in ICI therapeutic efficacy in numerous clinical cohorts^456–460^ and that optimal modulation of the gut microbiome is sufficient to strengthen the therapeutic response to ICIs in preclinical models.^43,461,462^

The particular gut microbiome seemingly has a positive influence on the effectiveness of ICIs via manipulating the tumor microenvironment in preclinical models. For example, antibiotic treatment prominently abolishes responses to ICIs in a mouse model with melanoma.^461^ However, gavage with *B. fragilis* or *Bifidobacterium* to GF or antibiotic-treated mice reverses the compromised efficacy of anti-CTLA-4 mAb and anti-PD-L1 mAb, respectively.^461,462^ Moreover, the anti-tumor effect by supplementation with *Bifidobacterium* alone is comparable to anti-PD-L1 treatment. The oral administration of *Bifidobacterium* in combination with anti-PD-L1 therapy almost completely eliminates melanoma.^461^ Colonization of mice with a mixture of 11-low-abundance bacteria (seven of which are *Bacteroidetes*) isolated from healthy human donors remarkably strengthens the ICI efficacy without causing side effects (such as inflammation).^43^ However, these promising experimental results are limited to animal studies.

The translational relevance of the above two studies to humans has been demonstrated in other work. Recurrent antibiotic exposure before or after the initiation of ICIs exerts detrimental impacts on immunotherapy efficacy. For example, among 249 patients with non-small-cell lung cancer (NSCLC), RCC, or urothelial cancer treated with PD-1 blockade, compared with patients who have never receipted antibiotics, those who have taken antibiotics are associated with unfavorable clinical outcomes, including more frequent tumor recurrence and significantly decreased OS (20.6 vs. 11.5 months, *P* < 0.001).^457^ Conversely, patients’ response rates to anti-PD-1 mAb can be elevated from 25 to 40% when refraining from antibiotics.^457^ Another study also highlights the analogous conclusion that disruption of the gut microbiome by antibiotics potentially compromises clinical responses to ICIs and thus results in poor PFS and OS in patients with advanced cancers.^463^ Notably, clinicians should not confuse these related studies with the pathological state requiring antibiotic treatment, as not all studies reveal the association between antibiotic exposure and unsatisfactory ICI efficacy.

In addition, specific bacterial species correlate with a much more favorable immunotherapy outcome. For example, NSCLC and RCC patients with favorable responses to anti-PD-1 treatment are characterized by enhanced fecal levels of *Akkermansia muciniphila* in comparison with nonresponders.^457^ Compared with mice colonized with the microbiome from nonresponder donors, GF mice receiving fecal microbiota transplantation (FMT) of material derived from responders (containing *A. muciniphila*) display much more advantageous therapeutic responses to anti-PD-1 mAb. Moreover, supplementation with *A. muciniphila* reverses the compromised efficacy in mice receiving the microbiome from nonresponders.^457^ Analogously, clinical responders with advanced melanoma are enriched in *F. prausnitzii* and *Clostridiales*, as well as with a high α-microbial diversity and significantly prolonged survival rate. In contrast, *Bacteroides* are abundant in clinical nonresponders.^458^ Interestingly, when analyzing the fecal microbiome derived from another metastatic melanoma patient cohort treated with anti-PD-1 immunotherapy, a significantly higher abundance of particular bacterial taxa (such as *B. longum*, *Collinsella aerofaciens*, and *Enterococcus faecium*) was detected in clinical responders than in nonresponders. Quantifying the proportion of ‘favorable’’ and ‘unfavorable’’ bacteria in patients reveals that a ratio of "favorable" microbiome to "unfavorable" microbiome >1.5 is highly associated with remarkably improved efficacy.^460^ The association between the gut microbiome and the response to anti-PD-1 immunotherapy in Chinese patients with NSCLC has also been revealed for the first time. Specifically, responders with significantly prolonged PFS are endowed with greater microbiome diversity as well as an increased abundance of *Alistipes putredinis*, *B.* *longum*, and *Prevotella copri*.^459^ Similarly, the gut microbiome potentially exerts a profound effect on the responses of HCC patients receiving anti-PD-1 mAb.^456^ Responders exhibit greater taxa richness and 20 responder-enriched species (such as *A. muciniphila* and *Ruminococcaceae* spp.) that are associated with carbohydrate catabolism and methanogenesis.^456^ Therefore, a causal role for the gut microbiome in modulating anti-tumor immunity and successful manipulation and exploitation of the gut microbiome with specific bacterial taxa can strengthen the therapeutic response in animal models. Notably, different ICI treatment plans correspond to the variation in the dominant gut microbiome among responders with metastatic melanoma, indicating that the relationship between the gut microbiome and every ICI therapy needs to be investigated one by one.^464^ Additionally, ICIs have been found to influence the gut microbiome composition in patients; dynamic alterations in the gut microbiomes of patients undergoing anti-PD-1 immunotherapy during different treatment cycles have been reported.^465^

These studies also elucidate the underlying mechanisms of the observed protection conferred by a favorable gut microbiome. *Bacteroides fragilis* can enhance the therapeutic response to anti-CTLA-4 mAb in melanoma via the IL-12-dependent Th1 immune response in the lymph nodes and facilitate the maturation and proliferation of intratumoral DCs.^462^ *Bifidobacterium* also augments CD8^+^ T cell responses to heighten anti-PD-1 effects.^460,461^ As exemplified by the Ki-67^+^CD8^+^ memory T cell induction, the microbiome may explain the improved clinical outcomes to anti-PD-1 immunotherapy.^459^ Similarly, the 11-strain consortium isolated from healthy individuals elicits IFN-γ^+^CD8^+^ T cells in a CD103^+^ DC-dependent manner, conferring enhanced efficacy of immunotherapy.^43^ *Akkermansia muciniphila* induces the release of DC-derived IL-12 and is correlated with increased recruitment of CCR9^+^CXCR3^+^CD4^+^ T cells into the tumor bed, accompanied by substantially reduced Foxp3^+^ Treg cells.^457^ Consequently, gut microbiome signatures conducive to the curative effects of ICIs are correlated with reinforced systemic anti-tumor immunity and intratumoral immune infiltrates. Notably, bacterial translocation resulting from impaired intestinal integrity is sufficient to shape and enhance the desirable anti-tumor immunity network by serving as MAMPs/pathogen-associated molecular patterns to interact with PRRs on innate immune cells, priming cross-reactive T cells against microbial antigens that resemble tumor antigens,^466^ but it also exerts detrimental effects on the onset and development of tumors, such as breast tumor.^415,416^ The completely opposite roles of systemic immune responses elicited by bacterial translocation in tumorigenesis and progression, as well as immunotherapy reflect the multifaceted relationship between inflammation and cancer.

The above studies in both preclinical models and human cohorts provide us with evidence that the microbial composition of the gut may have predictive clinical value for the therapeutic effect of ICIs. The potential confounders that may influence the research findings may also account for the phenomenon that specific microbiome signatures conducive to immunotherapy vary widely across the cohorts even in identical tumor backgrounds.^458,460^ The composition of the human microbiome is unique in each individual, and heritability accounts for merely 1.9% of the variation observed in distinct microbial communities. However, over 20% of interindividual microbiome variability is associated with environmental factors.^467^ Geographical- and ethnic-related characteristics, including sociodemographic, lifestyle, and dietary habits, are thought to partly explain these interindividual differences in gut microbiota composition.^468,469^ With regard to lifestyle, sleep deprivation can promote metabolic diseases by affecting gut microbial composition.^470^ Exercise appears to accelerate the production of microbial-derived SCFAs, along with an abundance of beneficial *A. muciniphila* and a decreased level of proinflammatory *Proteobacteria*.^471–473^ Chronic stress seems to dramatically alter the composition of the gut microbiome, with the expansion of inflammation-promoting bacteria as well as increased intestinal permeability.^474^ High-throughput in vitro screens of over 1000 marketed drugs against 40 representative human gut bacterial strains have revealed that 24% of host-targeted non-antibiotic drugs suppress the growth of at least one strain, indicating that the gastrointestinal side effects of these drugs potentially result from their effects on the gut microbiome.^475^ Non-antibiotic drugs, particularly proton-pump inhibitors,^476^ antidiabetic drugs such as metformin,^477^ antipsychotics,^475^ and nonsteroidal anti-inflammatory drugs^478^ are all determinants of interindividual heterogeneity in the composition and function of the gut microbiome. In addition, the microbiome in the intestinal mucosa differs from that in stool both quantitatively and compositionally.^479^ It is likely that misleading conclusions would be drawn if fecal microorganisms were considered to reflect the types of microorganisms in the gut, so it is essential to explore noninvasive techniques to collect samples from the gut mucosa. Moreover, sampling and analysis of the gut microbiome allows only a single time point to be studied, which means that differences in species or metabolite presence and/or abundance over time are neglected.^480^ Thus, the standardization of timing and intervals for microbiome profiling as well as microbial sampling sites could resolve some differences in the findings of various studies. Differences in collection, storage, sequencing, and analysis pipelines as well as experimental conditions can also explain these discrepancies.^481^ Microbial signals are intrinsic to every cohort but are functionally correlative, implying that function rather than specific bacterial species preferably describe and predict treatment effects.^481^ The search for microbial signals as predictors of tumor immunotherapy response requires in-depth investigation of microbial function and the integration of RNA-sequencing with metabolomics analysis to confirm the underlying pathways implicated in therapeutic efficacy. The synergistic effects between microbial structure (cell surface antigen, nucleic acids, and so on) and therapeutic efficacy warrant further exploration.^481^

### Potential microbial-based clinical therapeutics

Efforts are currently underway to identify optimal bacterial consortia and modulate the gut microbiome through accumulating experience from trials targeting nonmalignant disease or animal models with cancers, thereby potentiating therapeutic efficacy. Could a therapeutic answer to malignant tumors reside in the human gut? Could probiotics, FMT, or prebiotics become novel anti-tumor agents? An objective evaluation of the evidence is needed to determine whether the gut microbiome can be considered as a potential path forward for cancer treatment (Table 3).

**Table 3:** The microbial-based therapeutics

	Benefits	Drawbacks	Future focus
FMT	Significantly increased microbial diversity; robust colonization in the gut; competition against pathogens; limiting antibiotic resistance	Inaccessible and expensive; time-consuming for donor screening; FMT-related adverse events (injury induced by invasive medical operation, transmission of microbiome-mediated diseases)	Donor screening; methods for fecal pathogens detection; protocols for sample collection, preservation, and administration; use in different organs; high-quality clinical data with close follow-up; long-term safety investigation
Probiotics	Ease of use and accessibility	Inadequate supervision for quality control; difficulties in stable colonization; possibility of retarding the recovery of disordered gut; unknown mechanisms of behavior	Utilization of spores versus live bacteria; large-scale and convincing clinical trials; systemic assessment of safety and effectiveness; personalized treatment
Prebiotics	Available; inexpensive; easy to operate	The inability to significantly improve bacterial diversity; potential harm to health in some contexts	Appropriate application scenarios; safe and effective dose; reasonable duration; fiber with simple molecular structure vs. complex structure
Diets	Accessible; easy to operate; maintaining overall health	Difficult to alter ingrained dietary habits; difficulty in retaining expected and stabilized modulation of microbiota	Universal dietary guidelines; personalized diet recommendations; certain dietary ingredient vs. overall dietary pattern; persistent and stable control of the microbiome; selection of microorganisms to predict successful dietary interventions

### Fecal microbiota transplantation

FMT is defined as the transmission of the gut microbiome derived from healthy donors to unhealthy recipients through the digestive tract route, which aims to reestablish gut homeostasis or to provide a new balance in order to abrogate or ameliorate disease.^482^ FMT has been recognized as a standard treatment for recurrent CDI by official guidelines with a nearly 90% cure rate.^482,483^ Researchers are also increasingly realizing the potential of FMT in other noncancerous diseases, such as IBD,^484–486^ IBS,^487^ liver disease,^488^ and neuropsychiatric disorders.^489^ Hence, knowledge obtained from FMT in these diseases is also emerging evidence to indicate that FMT can be potentially employed in the management of cancer.

Compared with the limited clinical benefit of exploiting a single bacterial species, with FMT, an entire and sophisticated community is transplanted at once, which offers numerous potential applications and advantages.^482^ First, in the setting of overall community transplantation, the introduced microbiome is endowed with a more robust capacity to colonize the gut, as the recipient microbiome displays attenuated competitive exclusion.^490,491^ In addition, FMTs potentially dampen the spread of antibiotic resistance and enhance the time used to eliminate the remaining viable antibiotics, leading to effective and durable antibiotic concentrations in patients.^492^ In cases where antibiotics must be utilized in patients with severe infection, interventions such as autogenous fecal transplantation may be instrumental in restoring antibiotic-mediated disruption of the gut microbiome.

Although a laudable goal, owing to the imprecise definition of favorable fecal bacteria composition and the unidentified pathogenicity of detrimental microbiome, clinicians should not overlook the possibility that the OS benefit associated with FMT comes at the cost of other diseases that are transmitted from donor to recipient,^466,493^ including some mild and self-limited side effects (diarrhea, bloating, abdominal pain),^494,495^ obesity,^496^ infections,^497,498^ inflammation-induced carcinogenesis (the fecal microbiome from colorectal patients facilitates tumorigenesis in GF and carcinogenic mice),^499^ and even death.^500^

In principle, to make FMT more feasible in cancer therapy (including ICIs), several pivotal parameters should be taken into account. Initially, selection of an ideal donor remains a crucial issue, as preliminary evidence suggests that the gut microbiome profile of a donor is the determinant of the response rate to ICIs in mice with cancer.^43,457^ Several clinical trials in which FMT is being explored in cancer patients receiving ICI therapy are ongoing (NCT03353402 and NCT03341143), which take advantage of fecal materials from complete responder donors.^490^ However, as long as we can identify the microbiome that is conducive to cancer immunotherapy, clinicians should take advantage of balanced fecal microbiomes from healthy donors rather than dysbiotic microbiomes from patients.^501^ Recently, a randomized double-blind controlled trial indicated that the metabolic characteristics of donors can be delivered to the recipient to some extent, so the selection of metabolic conditions in donors should be considered one of the factors through which FMT can be optimized.^502^ Actually, a consensus on which particular bacterial species or the combination of bacteria are the optimal option for immune-potentiating effects has not been reached and warrants further investigation.^43,457,458,460,461^ Notably, low levels of bacterial taxa that coexist with the much more abundant species are potentially functionally pivotal.^43,503^ An additional method is implicated in an autologous FMT of fecal materials that need to be collected and preserved before an individual is sick, which necessitates large-scale fecal banking facilities. How to preserve FMT materials remains a methodological challenge. Frozen fecal products harbor lower bacterial diversity and are correlated with clinical efficacy in comparison to fresh material.^504,505^ The practice of preparing material for FMT in ambient air profoundly influences the viable microbial content, with disproportionately diminished levels of anaerobic commensals and thus a reduced capacity for the biosynthesis of pivotal anti-inflammatory metabolites.^505^ Clinical practice should take into account the administration methods of fecal microbiota when necessary. The routes of fecal infusion include oral administration (through nasogastric/nasojejunal tube or oral capsules), enema, and colonoscopy. FMT-mediated clinical efficacy for IBS patients is considered to vary with the administration route, with an increase in likelihood of improvement in colonoscopy or nasojejunal tube administration in contrast to multiple-dose oral encapsulated FMT.^506^ Nevertheless, a randomized controlled trial (RCT) indicated that there was no significant difference in the response rate to FMT between oral encapsulated products and delivery through colonoscopy, with oral capsulized FMT displaying decreased rates of minor adverse events and increased patient adherence.^507^ However, some questions remain unanswered, such as the optimal dose and interval for FMT, which may vary among different cancer populations.

### Microbial metabolite-mediated interventions

Even with a greater understanding of the molecular targets and biological significance of gut microbiome-derived metabolites, a wide array of pivotal parameters should be taken into account if we are to attempt to bridge current findings to therapeutically relevant contexts. Exogenous administration of microbiome-derived metabolites appears to be therapeutically feasible. As an example, an oral formulation of *B. fragilis*-derived PSA called SYMB-104 has passed preclinical testing and is being explored in patients with IBD.^61^ Indeed, treatment of autism spectrum disorder mice with candidate microbial metabolites, such as 5-aminopentanoic acid and taurine, ameliorates behavioral abnormalities and modulates neuronal excitability in the brain.^16^ Together, these results have shed light on the therapeutic potential of microbiome-derived molecules in animal models. Nevertheless, similar to all oral therapeutics, a considerable challenge is that metabolites are potentially assimilated or degraded before reaching the desired tissues and cells, triggering spontaneous side effects.^61^ Further studies are warranted to examine their long-term safety in humans.

### Probiotics

Probiotics are considered live microorganisms that putatively provide the host with health benefits when administered in adequate amounts. Probiotics such as *Lactobacillus* spp. and *Bifidobacterium* are frequently found in multitudinous products such as dietary supplements or drugs.^508^ It is at the forefront of the field to determine and exhibit the therapeutic or preventive effectiveness of probiotics in a broad range of diseases (such as sepsis in infants,^509^ infant colic,^510^ diarrhea,^511^ CDI,^512^ and infectious disease induced by pathogen *Staphylococcus aureus*^513^ as well as phenylketonuria,^514^) rather than attempting to enrich beneficial bacteria-derived metabolites. Theoretically, the stable engraftment of metabolites producing bacteria in the gut confers a long-term therapy choice, as a few front-loaded bacterial doses can supersede routine and repeated administration of a purified molecule to patients. Probiotics can transfer physiological concentrations of a molecule straight to the host, which eliminates the need for exogenous administration of high-dose chemical compounds and concomitant off-target effects.^61^ Interestingly, supplementation with pasteurized beneficial *A. muciniphila* exhibits a much more desirable therapeutic effect on ameliorating metabolic disorder compared to supplementation with live *A. muciniphila*.^28^ It remains unclear why pasteurized bacteria appear to be more advantageous in alleviating metabolic syndrome. Due to the infrequency of large, multicenter, randomized, placebo-controlled trials in distinct clinical scenarios and human subpopulations, clinical data from other studies are of variable quality, and some publications may exhibit bias towards studies harboring positive outcomes.^388^

The effects of exposure to probiotics on the enhanced anti-tumor immune response have been identified in a mouse model. For example, oral administration of probiotics Prohep (*LGG*, *E. coli Nissle* (*EcN*)1917 and heat-inactivated VSL #3 for a 1:1:1 mixture) results in significantly diminished tumor weight and volume and increased levels of anti-inflammatory bacteria such as *Prevotella* and *Oscillibacter*. Prohep serves as a negative regulator of inflammation and intratumoral angiogenesis by hampering intestinal Th17 cell development and downregulating the expression of angiogenic growth factors (such as ANG2 and VEGFA), thereby abolishing liver tumor progression.^515^ Probiotic *EcN*1917 can improve tumor-specific Teff cell infiltration and DC activation, significantly hampering HCC growth and metastasis.^516^ The suppressive effect of *Lactobacillus* probiotics on mammary cancer progression has been attributed to diminished IL-6 and enhanced IL-10 in serum and mammary gland cells.^429^ Short-term administration of *L. johnsonii* is associated with attenuated systemic leukocyte genotoxicity in a lymphoma mouse model.^517^ Similar benefits from supplementation with live *L. reuteri* have been observed in a leukemia mouse model.^518^ Supplementation with aerosolized probiotics remarkably potentiates immunity and chemotherapy activity against lung metastases in mice with melanoma, representing a potential delivery method for probiotics to modulate distant tumor metastases.^519^ Thus, certain live bacteria can activate numerous signaling pathways in the host to potentiate therapeutic effects through multiple mechanisms, thereby priming the host for further immunomodulation.^43^ Nevertheless, the overwhelming majority of current evidence connecting probiotics to extraintestinal tumor processes has been demonstrated in animal models. The effect of supplementation with probiotics has been tested in a clinical trial in patients with operable breast tumors before surgery (NCT03358511), which aims to evaluate alterations in the gut microbiome composition as well as changes in CD8^+^ T cell numbers in the tumor microenvironment.^466^ A previous prospective RCT comprising 138 patients with primary bladder cancer demonstrated that in patients managed by transurethral resection, the recurrence rate was significantly decreased following daily oral administration of *L. casei* preparation in contrast to placebo.^520^ The conclusive evidence of personalized probiotic interventions as negative regulators of sustained tumor progression in humans remains to be confirmed.

Despite the above positive effects, probiotics fail to hamper the proinflammatory response and cannot improve intestinal barrier function in the setting of DSS-induced intestinal barrier disruption. Conversely, it contributes to the increased production of IL-1β, IL-6, and TNF-α, indicating that probiotic supplementation is potentially risky under conditions of compromised barrier function.^521^ More surprisingly, compared to spontaneous post-antibiotic recovery, probiotic intervention exerts a notably delayed effect on the reestablishment of a diverse microbial ecosystem after antibiotic treatment and makes it difficult to completely return to its pre-antibiotic state. Conversely, autologous FMT can trigger the most rapid and complete recovery after perturbation.^479,522^ Hence, probiotics need to be carefully tested in clinical patients managed by long-term antibiotic treatment. Generally, the host with a diverse indigenous gut ecosystem tends to display an inherent resistance to repopulation by exogenous bacterial species, which enables the gut mucosal colonization of probiotic challenging.^479,522^ For most successfully colonized individuals, probiotics affect the overall community structure of the gut microbiome and host gene expression. After the cessation of bacterial replenishment, the essential microflora components in the feces quickly return to near normal levels, and only a small percentage of patients are continuously colonized by the bacteria in probiotics, suggesting that the effects of probiotics on mucosal community structure and intestinal transcriptome are transient and individualized.^479^ Thus, how to facilitate the stable colonization of exogenously therapeutic probiotics in the intestine is an essential issue to be solved. Because resource availability and the abundance of indigenous microbiome in the gut are pivotal factors controlling stable probiotic engraftment,^523,524^ an opportunity emerges for the context-specific tailoring of different probiotic strains, such as nutrients, to optimize the enrichment of probiotics and downstream activity.^525^ For instance, administration of prebiotics such as inulin confers substrates for certain commensals and thereby triggers a divinable phylogenetic and functional reconstruction of the gut microbiome.^526,527^ Future studies are warranted to investigate the underlying mechanisms of the activities of various probiotic strains in vivo in order to predict microbial alterations in the gut after probiotic intervention. Human feeding studies are also needed to confirm their relevance, safety, and effectiveness before they can be translated to practical and individualized nutrition advice.

Another strategy for microbiome-based therapies is to utilize synthetically engineered microorganisms with modified bacterial functions or introduced entire new genes. An engineered *EcN* can express and secrete Phe-metabolizing enzymes that are activated in the gut anaerobic environment and metabolize Phe into excreted Hippurate, thus treating phenylketonuria in both monkeys and mice models. This strain has been employed in clinical trials currently underway to determine the effective dose in the human body.^514^ Similarly, another engineered probiotic, *EcN*, facilitates the conversion of NH3 into l-arginine to mitigate hyperammonemia symptoms. This strain has been moved into a phase 1 clinical study and has exhibited secure and dose-dependent metabolic activity in vivo, indicating its therapeutic potential in hyperammonemia disorders, including urea cycle disorders and hepatic encephalopathy.^528^ A *Bacteroides* strain modified to harbor a gene cluster conducive to using porphyrin has the capacity to colonize stably in mice with a porphyran-supplemented diet, which effectively confers a unique metabolic niche for the exogenous microbiome and presents a potential strategy to potentiate the efficacy of the target strain.^524^ Nevertheless, certain obstacles remain prior to the implementation of engineered bacterium therapy in clinical practice. As a form of gene therapy, the imminent risk is that the microbiome has the potential to transfer human genes to additional bacteria in the host, thus triggering unpredictable consequences. Additional evidence will emerge as the current set of clinical trials wraps up in the next few years.^529^

### Diet and prebiotics

Individualized dietary modification is an ideal strategy to maintain healthy physiology in the host. HFD correlates with increased *Alistipes*, *Prevotella*, and reduced α-diversity,^97,530^ while a high-fiber diet is associated with enhanced butyrate-producing bacteria.^527^ The effects of additional dietary types on specific gut microbiomes have also received much attention. For example, a ketogenic diet (KD) emphasizes the consumption of very low CHO consumption (5 to 10% of total caloric intake) and is sufficient to enhance ketone production. KD was initially developed as a therapeutic intervention for refractory childhood epilepsy.^531^ The enrichment of *Akkermansia* and *Parabacteroides* associated with KD can mediate neuroprotective and anti-seizure effects.^532,533^ A high salt diet potentially worsens colitis in mice and accelerates the occurrence of hypertension and cardiovascular disease, which is associated with alterations in gut immune homeostasis and gut microbiome composition, including a reduced relative abundance of *Lactobacillus* spp. and an increased *Firmicutes*/*Bacteroidetes* ratio.^279,534^ Such alterations have also been revealed to exert impacts on the immune response and metabolism in mice and humans. Considering these results, dietary modulation represents an additional avenue through which cancer treatment can be improved.^181,535^

However, owing to the deficiency of convincing clinicians and patients of normalized dietary guidelines, patients can be nonadherent to prespecified dietary recommendations.^394^ Additionally, short and intense modulation of dietary patterns can elicit rapid and reproducible alterations in the gut microbiome, but it is relatively difficult to modulate the gut microbiome community structure and function through long-term dietary intervention.^536^ Another consideration is that merely a fraction of the overall community will respond sufficiently to diet-mediated stimuli; thus, greater understanding is needed in terms of whether such a response would trigger anticipated health outcomes in the host.^537^ Another unresolved problem is how to identify the pivotal diet-responsive microbiome that can serve as the predictor of clinically successful dietary interventions.

In addition, prebiotics are considered substrates (such as oligofructose and inulin) that selectively support the growth and activity of one or a few putatively beneficial gut microbiomes to exert a beneficial impact on the host.^538^ Nevertheless, prebiotic modulation potentially fails to noticeably increase the total community diversity of the gut microbiome or additional bacterial species implicated in complementary metabolic or/and immune functions.^490^

A promising role of prebiotics in inhibiting cancers in a microbiome-dependent manner has been consistently demonstrated across studies of animal models. For example, administration of inulin-type fructans participates in restoring the gut microbiome and AMP expression, thereby leading to prolonged survival and subdued cancer proliferation and cachexia in leukemia mice.^518^ In another mouse model with liver cancer receiving inulin-type fructans (ITFs), the proliferation of liver cancer cells is counteracted through ITF-fermented propionate generation.^539^ Astoundingly, long-term consumption of dietary soluble fibers (such as inulin, pectin, and fructooligosaccharide) that have long been regarded as “prebiotics,” actually triggers cholestasis and icteric HCC in 40% of T5KO dysbiotic mice as well as in additional dysbiotic mice model deficiency in TLR4 or NLRC4. Furthermore, such HCC is not observed in GF- or antibiotic-treated mice but can be transmissible to healthy WT mice. The depletion of butyrate-producing bacteria or inhibition of gut fermentation results in a significant reduction in gut SCFA and protection against such HCC, revealing that gut dysbiosis exerts a decisive role in the onset and development of HCC.^32^ Therefore, in the context of gut dysbiosis, long-term supplementation of inulin or/and butyrate potentially leads to a more disconcerting microbial composition and function that confers a hazard to the health of the host. Although this study was conducted in mice and failed to be directly analogized to humans, it indeed backs up the underlying implications that, in patients with gut dysbiosis, enrichment of foods with prebiotic fibers may be unsound due to their dysregulated fermentation. Single prebiotics fail to provide metabolic benefits for the balanced growth of the overall intestinal community. The conditions that determine whether prebiotics play a beneficial or detrimental part in the host may partly depend on particular application scenarios and the real demand of customers. Further studies are warranted to comprehensively analyze the pleiotropic impacts of prebiotics on the host.

## Conclusion

The intricate and diverse characteristics of the gut microbiome enable it to be an appealing target for therapeutic manipulation in diverse settings. The microbiome exerts effects on the host—in particular, it profoundly influences tumorigenesis and the development of various tumors through microbiome-derived metabolites or via direct modulation of immune and metabolism in the host. Elevating our understanding of how the microbiome and its metabolites affect host immunity will advance our capacity to provide well-founded microbial-based therapeutics.

## Supplementary information

Supplementary Materials
