# Supplementary material for: Demystifying the manipulation of host immunity, metabolism, and extraintestinal tumors by the gut microbiome
Source: Signal Transduct Target Ther. 2019 Oct 12;4:41. doi: 10.1038/s41392-019-0074-5 (PMC6799818; doi:10.1038/s41392-019-0074-5)
Supplement: Supplementary file 1 — Supplementary Materials [file 41392_2019_74_MOESM1_ESM.docx]

Supplementary Materials for

Demystifying the manipulation of host immunity, metabolism, and extraintestinal tumors by the gut microbiome

Ziying Zhang, Haosheng Tang, Peng Chen, Hui Xie, Yongguang Tao

Correspondence to: taoyong@csu.edu.cn

**This PDF file includes:**

Tables S1 to S2

| Figure. S1. Effects of microbial-derived SCFAs on the host | | | | |
| --- | --- | --- | --- | --- |
| Disease Relevance | Metabolite | Effects | Mechanisms | Ref |
| Protective against colitis | SCFAs | Induce protective Th17 cells | Depend on HDAC inhibitor activity | [1] |
|  |  | Promote Th1 cell-derived IL-10 production | Activate GPR43 and upregulate Blimp-1 expression | [2] |
|  |  | Enhance plasma B cell differentiation and Ab production | Facilitate energy synthesis for Ab production | [3] |
|  |  |  | Upregulate differentiation-related genes through HDAC inhibitor activity | [3] |
|  |  |  | Accelerate B cell differentiation via AFF and ALDH1a2 production by DCs | [4] |
|  | Propionate | Foxp3^+^/IL-10^+^ Tregs expansion | Signals through GPR43 on naive T cell | [5,6] |
|  | Butyrate | Foxp3+/IL-10+ Tregs expansion | Activates GPR109A on DCs and macrophages | [7]7 |
|  |  |  | Suppresses HDAC at the Foxp3 promoter and at CNS1 | [8,9] |
|  |  |  | Potentiates TGF-β1 expression in IECs | [10] |
|  |  | Facilitates M2 macrophage polarization and function | Decreases the secretion of NO, IL-6, IL-12 and TNF | [11] |
|  |  |  | Upregulates Arg1 to modulate metabolic reprogramming | [12] |
| Colonization against pathogens | Butyrate | Enhances AMPs production | Inhibits HDAC3 in macrophage | [13] |
|  |  |  | Activates GPR43-mediated mTOR and STAT3 in IECs | [14] |
|  |  | Fosters hypoxic intestinal microenvironment | Activates PPAR-γ | [15,16] |
|  |  | Disturbs pathogen pH homeostasis | Facilitates intracellular acidification of Salmonella | [17] |
| Enhance barrier integrity | SCFAs | Activates NLRP3 and IL-18 secretion | Signal though GPR43 on IECs | [18,19] |
|  |  | Facilitate Mucin 2 secretion | Activate NLRP6 | [20,21] |
|  | Butyrate | Enhances apical junctional protein expression | Inhibits HDAC | [22] |
|  |  |  | Activates HIF-1 in IECs | [23] |
| Modulate CNS disease | SCFAs | Potentiate BBB integrity | Upregulate the tight junction protein occludin | [24] |
|  |  | Maintain CNS immune cells homeostasis | Stimulate microglial maturation | [25] |
|  | Propionate | Inhibits inflammation in BBB | Protects the BBB from oxidative stress | [26] |
|  |  |  | Signals through FFAR3 on the brain-endothelium-expressed to suppress TLR | [26] |
| Protective against autoimmune diabetes | Acetate | Inhibits autoreactive T cells | diminishes marginal zone B (MZB) cells | [27,28] |
|  | Butyrate | Tregs expansion | Inhibits HDAC | [26,27] |
|  |  | Pancreatic regulatory macrophages and Tregs expansion | IL-22-mediated β-defensin 14 secretion by pancreatic endocrine cells | [26,27] |
| Improve autoimmune lupus | SCFAs | Ameliorate pDCs, IFN-I pathways | Suppress L. reuteri overgrowth | [29] |
| Protective against influenza | SCFAs | Enhance anti-viral CD8^+^ T cell responses | Boost T cell metabolism | [30] |
|  |  | Dampen neutrophils-mediated deleterious immunopathology | Reduce the chemokine CXCL1 produced by macrophages | [30] |
| Maintains bone homeostasis | Butyrate | Increases Wnt10b production by bone marrow-resident CD8^+^ T cells | Increases TGF-β produced by Tregs | [31] |
|  | Propionate and butyrate | Downregulate essential osteoclast genes | Enhance glycolysis in osteoclasts | [32] |
| ， | Acetate and butyrate | Decrease appetite and energy intake | Stimulate GLP-1 and PYY generation | [18,33] |
|  | Acetate | Triggers anorectic phenotype in the hypothalamus | Inhibits 5 -AMP-activated kinase AMPK | [34,35] |
|  | Butyrate | Activates IGN | cAMP-mediated IGN gene upregulation | [36] |
|  | Propionate | Activates IGN | Signals through GPR41 on peripheral nerves of the portal vein | [36] |
|  | Butyrate | Increases energy expenditure and lipid oxidation | Enhances catabolism-associated genes | [37] |
|  |  |  | Inhibits HDAC3 | [37] |
|  | Butyrate | Mitigates endotoxaemia and atherosclerosis | Enhances fatty acid utilization | [38] |
|  | Butyrate | Involved in thermoregulation | Enhances the thermogenic capacity of brown adipose tissue | [39] |

| **Table S2.** Effects of microbial TRP metabolites on the host | | | | |  |
| --- | --- | --- | --- | --- | --- |
| Disease relevance | Metabolites | Effect | Mechanism | Ref |  |
| Protective against colitis | ILA | Promotes the development of CD4^+^ CD8αα double-positive IELs | Activates AHR in CD4^+^ IELs | [40] |  |
|  | ICA | Decreases inflammatory cytokine production | Elicits IFN-I response in IECs | [41] |  |
| Colonization resistance against pathogens | IAA, IAId | Induce IL-22 secretion by ILC3 | Activate AHR | [42] |  |
|  | Indole | Mitigates Salmonella virulence and invasiveness | Unknown | [43,44] |  |
| Fortify barrier integrity | Indole-3-carbaldehyde | Induce IL-22 secretion by ILC3 | Activates AHR | [45] |  |
|  | Indole | Facilitates apical junction proteins expression | Independent of AHR | [46] |  |
|  | IA, IPA | Enhances Muc2 expression by goblet cells | Independent of AHR | [47,48] |  |
|  | IPA | Diminishes intestinal permeability | Activates PXR or TLR4 on IECs | [49] |  |
| Mitigate EAE | I3S, IPA,IAId | Suppress NF-_K_B through SOCS2 | Activate AHR in astrocytes | [50,51] |  |
|  | TRP metabolites | Dampen pro-inflammatory activity in astrocytes | Activate AHR and TGF-α, suppress VEGF-B in microglia | [52] |  |
| Protective against autoimmune diabetes | I3A | Pancreatic regulatory macrophages and Treg cells expansion | IL-22-mediated β-defensin 14 secretion by pancreatic endocrine cells | [53] |  |
| Modulate hepatic immunity | Indole | Mitigates LPS-induced hepatic inflammation | Suppresses NF-_K_B | [54] |  |
|  | Indole-3-acetic acid | diminishes pro-inflammatory cytokines production in macrophages | Suppresses NF-κB | [55] |  |
|  | IAA | Induces IL-22 production by ILC3s and maintains REG3G expression | Signals through AHR | [55] |  |
| Modulate host metabolism | Indole, IAA | Increase GLP-1 secretion, improve glucolipid metabolism | Activates AHR | [57] |  |
|  | Indole | Increases GLP-1 secretion | Suppresses K^+^ channel | [57] |  |
|  | I3A | Mitigates lipogenesis in hepatocytes | Activates AHR | [58] |  |
| Modulate IBS | Tryptamine | Accelerates colonic fluid secretion and gastrointestinal motility | Signals through serotonin receptor-4 on gut epithelium | [59,60] | |

**References**

1. Park, J. *et al.* Short-chain fatty acids induce both effector and regulatory T cells by suppression of histone deacetylases and regulation of the mTOR-S6K pathway. *Mucosal immunology* **8**, 80-93, doi:10.1038/mi.2014.44 (2015).
2. Sun, M. *et al.* Microbiota-derived short-chain fatty acids promote Th1 cell IL-10 production to maintain intestinal homeostasis. *Nature communications* **9**, 3555, doi:10.1038/s41467-018-05901-2 (2018).
3. Kim, M., Qie, Y., Park, J. & Kim, C. H. Gut Microbial Metabolites Fuel Host Antibody Responses. *Cell host & microbe* **20**, 202-214, doi:10.1016/j.chom.2016.07.001 (2016).
4. Wu, W. *et al.* Microbiota metabolite short-chain fatty acid acetate promotes intestinal IgA response to microbiota which is mediated by GPR43. *Mucosal immunology* **10**, 946-956, doi:10.1038/mi.2016.114 (2017).
5. Maslowski, K. M. *et al.* Regulation of inflammatory responses by gut microbiota and chemoattractant receptor GPR43. *Nature* **461**, 1282-1286, doi:10.1038/nature08530 (2009).
6. Smith, P. M. *et al.* The microbial metabolites, short-chain fatty acids, regulate colonic Treg cell homeostasis. *Science (New York, N.Y.)* **341**, 569-573, doi:10.1126/science.1241165 (2013).
7. Singh, N. *et al.* Activation of Gpr109a, receptor for niacin and the commensal metabolite butyrate, suppresses colonic inflammation and carcinogenesis. *Immunity* **40**, 128-139, doi:10.1016/j.immuni.2013.12.007 (2014).
8. Arpaia, N. *et al.* Metabolites produced by commensal bacteria promote peripheral regulatory T-cell generation. *Nature* **504**, 451-455, doi:10.1038/nature12726 (2013).
9. Furusawa, Y. *et al.* Commensal microbe-derived butyrate induces the differentiation of colonic regulatory T cells. *Nature* **504**, 446-450, doi:10.1038/nature12721 (2013).
10. Martin-Gallausiaux, C. *et al.* Butyrate produced by gut commensal bacteria activates TGF-beta1 expression through the transcription factor SP1 in human intestinal epithelial cells. *Sci Rep* **8**, 9742, doi:10.1038/s41598-018-28048-y (2018).
11. Chang, P. V., Hao, L., Offermanns, S. & Medzhitov, R. The microbial metabolite butyrate regulates intestinal macrophage function via histone deacetylase inhibition. *Proceedings of the National Academy of Sciences of the United States of America* **111**, 2247-2252, doi:10.1073/pnas.1322269111 (2014).
12. Scott, N. A. *et al.* Antibiotics induce sustained dysregulation of intestinal T cell immunity by perturbing macrophage homeostasis. *Sci Transl Med* **10**, doi:10.1126/scitranslmed.aao4755 (2018).
13. Schulthess, J. *et al.* The Short Chain Fatty Acid Butyrate Imprints an Antimicrobial Program in Macrophages. *Immunity* **50**, 432-445.e437, doi:10.1016/j.immuni.2018.12.018 (2019).
14. Zhao, Y. *et al.* GPR43 mediates microbiota metabolite SCFA regulation of antimicrobial peptide expression in intestinal epithelial cells via activation of mTOR and STAT3. *Mucosal immunology* **11**, 752-762, doi:10.1038/mi.2017.118 (2018).
15. Byndloss, M. X. *et al.* Microbiota-activated PPAR-gamma signaling inhibits dysbiotic Enterobacteriaceae expansion. *Science (New York, N.Y.)* **357**, 570-575, doi:10.1126/science.aam9949 (2017).
16. Jacobson, A. *et al.* A Gut Commensal-Produced Metabolite Mediates Colonization Resistance to Salmonella Infection. *Cell host & microbe* **24**, 296-307.e297, doi:10.1016/j.chom.2018.07.002 (2018).
17. Macia, L. *et al.* Metabolite-sensing receptors GPR43 and GPR109A facilitate dietary fibre-induced gut homeostasis through regulation of the inflammasome. *Nature communications* **6**, 6734, doi:10.1038/ncomms7734 (2015).
18. Nowarski, R. *et al.* Epithelial IL-18 Equilibrium Controls Barrier Function in Colitis. *Cell* **163**, 1444-1456, doi:10.1016/j.cell.2015.10.072 (2015).
19. Birchenough, G. M., Nystrom, E. E., Johansson, M. E. & Hansson, G. C. A sentinel goblet cell guards the colonic crypt by triggering Nlrp6-dependent Muc2 secretion. *Science (New York, N.Y.)* **352**, 1535-1542, doi:10.1126/science.aaf7419 (2016).
20. Ranson, N. *et al.* Nod-Like Receptor Pyrin-Containing Protein 6 (NLRP6) Is Up-regulated in Ileal Crohn's Disease and Differentially Expressed in Goblet Cells. *Cell Mol Gastroenterol Hepatol* **6**, 110-112.e118, doi:10.1016/j.jcmgh.2018.03.001 (2018).
21. Mathewson, N. D. *et al.* Gut microbiome-derived metabolites modulate intestinal epithelial cell damage and mitigate graft-versus-host disease. *Nat Immunol* **17**, 505-513, doi:10.1038/ni.3400 (2016).
22. Fachi, J. L. *et al.* Butyrate Protects Mice from Clostridium difficile-Induced Colitis through an HIF-1-Dependent Mechanism. *Cell Rep* **27**, 750-761.e757, doi:10.1016/j.celrep.2019.03.054 (2019).
23. Braniste, V. *et al.* The gut microbiota influences blood-brain barrier permeability in mice. *Sci Transl Med* **6**, 263ra158, doi:10.1126/scitranslmed.3009759 (2014).
24. Chen, R. *et al.* Transplantation of Fecal Microbiota Rich in Short Chain Fatty Acids and Butyric Acid Treat Cerebral Ischemic Stroke by Regulating Gut Microbiota. *Pharmacological research*, 104403, doi:10.1016/j.phrs.2019.104403 (2019).
25. Hoyles, L. *et al.* Microbiome-host systems interactions: protective effects of propionate upon the blood-brain barrier. *Microbiome* **6**, 55, doi:10.1186/s40168-018-0439-y (2018).
26. Marino, E. *et al.* Gut microbial metabolites limit the frequency of autoimmune T cells and protect against type 1 diabetes. *Nat Immunol* **18**, 552-562, doi:10.1038/ni.3713 (2017).
27. Miani, M. *et al.* Gut Microbiota-Stimulated Innate Lymphoid Cells Support beta-Defensin 14 Expression in Pancreatic Endocrine Cells, Preventing Autoimmune Diabetes. *Cell Metab* **28**, 557-572.e556, doi:10.1016/j.cmet.2018.06.012 (2018).
28. Zegarra-Ruiz, D. F. *et al.* A Diet-Sensitive Commensal Lactobacillus Strain Mediates TLR7-Dependent Systemic Autoimmunity. *Cell host & microbe* **25**, 113-127.e116, doi:10.1016/j.chom.2018.11.009 (2019).
29. Trompette, A. *et al.* Dietary Fiber Confers Protection against Flu by Shaping Ly6c(-) Patrolling Monocyte Hematopoiesis and CD8(+) T Cell Metabolism. *Immunity* **48**, 992-1005.e1008, doi:10.1016/j.immuni.2018.04.022 (2018).
30. Ludwig, I. S. *et al.* Lactobacillus rhamnosus GG-Derived Soluble Mediators Modulate Adaptive Immune Cells. *Frontiers in immunology* **9**, 1546, doi:10.3389/fimmu.2018.01546 (2018).
31. Lucas, S. *et al.* Short-chain fatty acids regulate systemic bone mass and protect from pathological bone loss. *Nature communications* **9**, 55, doi:10.1038/s41467-017-02490-4 (2018).
32. Li, Z. *et al.* Butyrate reduces appetite and activates brown adipose tissue via the gut-brain neural circuit. *Gut* **67**, 1269-1279, doi:10.1136/gutjnl-2017-314050 (2018).
33. De Vadder, F. *et al.* Microbiota-generated metabolites promote metabolic benefits via gut-brain neural circuits. *Cell* **156**, 84-96, doi:10.1016/j.cell.2013.12.016 (2014).
34. Whitt, J. *et al.* Disruption of Epithelial HDAC3 in Intestine Prevents Diet-Induced Obesity in Mice. *Gastroenterology* **155**, 501-513, doi:10.1053/j.gastro.2018.04.017 (2018).
35. Kasahara, K. *et al.* Interactions between Roseburia intestinalis and diet modulate atherogenesis in a murine model. *Nat Microbiol* **3**, 1461-1471, doi:10.1038/s41564-018-0272-x (2018).
36. Kamareddine, L., Robins, W. P., Berkey, C. D., Mekalanos, J. J. & Watnick, P. I. The Drosophila Immune Deficiency Pathway Modulates Enteroendocrine Function and Host Metabolism. *Cell Metab* **28**, 449-462.e445, doi:10.1016/j.cmet.2018.05.026 (2018).
37. Li, B. *et al.* Microbiota Depletion Impairs Thermogenesis of Brown Adipose Tissue and Browning of White Adipose Tissue. *Cell Rep* **26**, 2720-2737.e2725, doi:10.1016/j.celrep.2019.02.015 (2019).
38. Scheiman, J. *et al.* Meta-omics analysis of elite athletes identifies a performance-enhancing microbe that functions via lactate metabolism. *Nat Med*, doi:10.1038/s41591-019-0485-4 (2019).
39. Erny, D. *et al.* Host microbiota constantly control maturation and function of microglia in the CNS. *Nat Neurosci* **18**, 965-977, doi:10.1038/nn.4030 (2015).
40. Cervantes-Barragan, L. *et al.* Lactobacillus reuteri induces gut intraepithelial CD4(+)CD8alphaalpha(+) T cells. *Science (New York, N.Y.)* **357**, 806-810, doi:10.1126/science.aah5825 (2017).
41. Swimm, A. *et al.* Indoles derived from intestinal microbiota act via type I interferon signaling to limit graft-versus-host disease. *Blood* **132**, 2506-2519, doi:10.1182/blood-2018-03-838193 (2018).
42. Agus, A., Planchais, J. & Sokol, H. Gut Microbiota Regulation of Tryptophan Metabolism in Health and Disease. *Cell host & microbe* **23**, 716-724, doi:10.1016/j.chom.2018.05.003 (2018).
43. Kumar, A. & Sperandio, V. Indole Signaling at the Host-Microbiota-Pathogen Interface. *mBio* **10**, doi:10.1128/mBio.01031-19 (2019).
44. Kohli, N. *et al.* The microbiota metabolite indole inhibits Salmonella virulence: Involvement of the PhoPQ two-component system. *PLoS One* **13**, e0190613, doi:10.1371/journal.pone.0190613 (2018).
45. Teng, Y. *et al.* Plant-Derived Exosomal MicroRNAs Shape the Gut Microbiota. *Cell host & microbe* **24**, 637-652.e638, doi:10.1016/j.chom.2018.10.001 (2018).
46. Roager, H. M. & Licht, T. R. Microbial tryptophan catabolites in health and disease. *Nature communications* **9**, 3294, doi:10.1038/s41467-018-05470-4 (2018).
47. Wlodarska, M. *et al.* Indoleacrylic Acid Produced by Commensal Peptostreptococcus Species Suppresses Inflammation. *Cell host & microbe* **22**, 25-37.e26, doi:10.1016/j.chom.2017.06.007 (2017).
48. Birchenough, G. & Hansson, G. C. Bacteria Tell Us How to Protect Our Intestine. *Cell host & microbe* **22**, 3-4, doi:10.1016/j.chom.2017.06.011 (2017).
49. Venkatesh, M. *et al.* Symbiotic bacterial metabolites regulate gastrointestinal barrier function via the xenobiotic sensor PXR and Toll-like receptor 4. *Immunity* **41**, 296-310, doi:10.1016/j.immuni.2014.06.014 (2014).
50. Rothhammer, V. *et al.* Microglial control of astrocytes in response to microbial metabolites. *Nature* **557**, 724-728, doi:10.1038/s41586-018-0119-x (2018).
51. Rothhammer, V. *et al.* Type I interferons and microbial metabolites of tryptophan modulate astrocyte activity and central nervous system inflammation via the aryl hydrocarbon receptor. *Nat Med* **22**, 586-597, doi:10.1038/nm.4106 (2016).
52. Wekerle, H. Brain inflammatory cascade controlled by gut-derived molecules. *Nature* **557**, 642-643, doi:10.1038/d41586-018-05113-0 (2018).
53. Miani, M. *et al.* Gut Microbiota-Stimulated Innate Lymphoid Cells Support beta-Defensin 14 Expression in Pancreatic Endocrine Cells, Preventing Autoimmune Diabetes. *Cell Metab* **28**, 557-572.e556, doi:10.1016/j.cmet.2018.06.012 (2018).
54. Beaumont, M. *et al.* The gut microbiota metabolite indole alleviates liver inflammation in mice. *Faseb j*, fj201800544, doi:10.1096/fj.201800544 (2018).
55. Krishnan, S. *et al.* Gut Microbiota-Derived Tryptophan Metabolites Modulate Inflammatory Response in Hepatocytes and Macrophages. *Cell Rep* **23**, 1099-1111, doi:10.1016/j.celrep.2018.03.109 (2018).
56. Hendrikx, T. *et al.* Bacteria engineered to produce IL-22 in intestine induce expression of REG3G to reduce ethanol-induced liver disease in mice. *Gut*, doi:10.1136/gutjnl-2018-317232 (2018).
57. Williams, B. B. *et al.* Discovery and characterization of gut microbiota decarboxylases that can produce the neurotransmitter tryptamine. *Cell host & microbe* **16**, 495-503, doi:10.1016/j.chom.2014.09.001 (2014).
58. Krishnan, S. *et al.* Gut Microbiota-Derived Tryptophan Metabolites Modulate Inflammatory Response in Hepatocytes and Macrophages. *Cell Rep* **23**, 1099-1111, doi:10.1016/j.celrep.2018.03.109 (2018).
59. Bhattarai, Y. *et al.* Gut Microbiota-Produced Tryptamine Activates an Epithelial G-Protein-Coupled Receptor to Increase Colonic Secretion. *Cell host & microbe* **23**, 775-785.e775, doi:10.1016/j.chom.2018.05.004 (2018).
60. Cryan, J. F., Clarke, G., Dinan, T. G. & Schellekens, H. A Microbial Drugstore for Motility. *Cell host & microbe* **23**, 691-692, doi:10.1016/j.chom.2018.05.020 (2018).
